# Native language experience shapes pre‐attentive foreign tone processing and guides rapid memory trace build‐up: An ERP study

**DOI:** 10.1111/psyp.14042

**Published:** 2022-03-16

**Authors:** Sabine Gosselke Berthelsen, Merle Horne, Yury Shtyrov, Mikael Roll

**Affiliations:** ^1^ Department of Linguistics and Phonetics Lund University Lund Sweden; ^2^ Department of Nordic Studies and Linguistics University of Copenhagen Copenhagen Denmark; ^3^ Center of Functionally Integrative Neuroscience Aarhus University Aarhus Denmark; ^4^ Institute for Cognitive Neuroscience HSE University Moscow Russia

**Keywords:** ERPs, L1–L2 similarity, pre‐attentive lexicality effect, second‐language acquisition, tone perception

## Abstract

Language experience, particularly from our native language (L1), shapes our perception of other languages around us. The present study examined how L1 experience moulds the initial processing of foreign (L2) tone during acquisition. In particular, we investigated whether learners were able to rapidly forge new neural memory traces for novel tonal words, which was tracked by recording learners’ ERP responses during two word acquisition sessions. We manipulated the degree of L1–L2 familiarity by comparing learners with a nontonal L1 (German) and a tonal L1 (Swedish) and by using tones that were similar (fall) or dissimilar (high, low, rise) to those occurring in Swedish. Our results indicate that a rapid, pre‐attentive memory trace build‐up for tone manifests in an early ERP component at ~50 ms but only at particularly high levels of L1–L2 similarity. Specifically, early processing was facilitated for an L2 tone that had a familiar pitch shape (fall) and word‐level function (inflection). This underlines the importance of these L1 properties for the early processing of L2 tone. In comparison, a later anterior negativity related to the processing of the tones’ grammatical content was unaffected by native language experience but was instead influenced by lexicality, pitch prominence, entrenchment, and successful learning. Behaviorally, learning effects emerged for all learners and tone types, regardless of L1–L2 familiarity or pitch prominence. Together, the findings suggest that while L1‐based facilitation effects occur, they mainly affect early processing stages and do not necessarily result in more successful L2 acquisition at behavioral level.

## Abstract 2

Our findings add important evidence that contributes to answering the open question of how similarity between native and target language influences target language processing and acquisition. We found facilitative effects of similarity only at pre‐attentive levels and only when the degree of similarity was high. Late processing and successful acquisition, on the other hand, were unaffected by the target words’ similarity to native language properties.

## 1 INTRODUCTION

The sounds and rules of our native language influence how we perceive a foreign language when we are first exposed to it. If something functions as a lexical or grammatical cue in our native language (L1), we are likely to pay more attention to this type of information in a second language (L2) (Ellis & Sagarra, 2011). This is also argued to be the case with tone. For instance, listeners pay particular attention to pitch movement in a foreign language if their native language makes use of pitch movements to distinguish meaning (Gandour, 1983). The present study investigates whether L1 experience also affects the learners’ neural responses to novel tone information. Furthermore, since in languages where tone is related to grammar, it can be argued to be more subtle and potentially less salient, we also addressed the role of L1 background in L2 acquisition for listeners whose native language uses tones to convey grammatical rather than lexical information.

### 1.1 Tone

Between 40 and 70% of the world’s languages are tonal (Maddieson, 2013; Yip, 2002), that is, they include pitch gestures that are added onto syllables or words to distinguish lexical items (lexical tone) or to add or strengthen grammatical information (grammatical tone). In a language like Mandarin, tone has a strongly lexical function such that, for instance, the syllable *ma* produced with a high level tone (T1) translates to “mother,” while it means “horse” when produced with a fall‐rise pitch contour (T3). In a language like Somali, on the other hand, tone has a strongly grammatical function and, for example, the change from a non‐high to a high tone on the ultimate vowel in a noun translates to a shift from nominative to genitive case (Banti, 1989). While both uses of tone contribute substantially to the language system, it might be argued that lexical tone is more salient and more strictly necessary than tone with a purely grammatical function. Unlike lexical tone languages, where tone can be realized on almost every syllable, in grammatical tone languages tones only occur in morphosyntactically licenced positions. Further, lexical content in language is more fundamental than grammatical inflections, as suggested by letter detection studies where readers pay more attention to lexical word stems than grammatical affixes (Koriat et al., 1991; Koriat & Greenberg, 1991). Carried over to the tonal domain, misuse or lack of tone might hinder communication more strongly for lexical tone than for grammatical tone. Consequentially, speakers of languages with mainly grammatical tone may rely slightly less on the tones, although tone is undoubtedly still highly entrenched.

Another important classification of tone is related to the tones’ acoustic features. In this respect, tone languages are crudely divided into register and contour tone languages. In register tone languages, tones are predominantly distinguished with respect to pitch level (e.g., Yoruba: high, mid, low), while contour tone languages distinguish tones according to pitch movement as well as pitch level (e.g., Cantonese: high, mid, low, mid‐rise, low‐rise, fall). Tone is perhaps most well‐known in the East‐Asian languages. Still, it also plays a vital role in many African and Native American languages and even in several European languages. While some tone languages are small or even facing extinction, others are thriving. The language with the largest number of native speakers in the world is the tonal Mandarin Chinese (>920 million native language (L1) speakers, Eberhard et al., 2020).

One of the European languages to feature tone is Swedish. Swedish tones are traditionally described as pitch accents. However, the concept of pitch accent languages has recently been questioned (e.g., Hyman, 2009, 2016). Therefore, we will briefly describe the tone system in its current state, focusing on the tones’ important interaction with grammatical processes. Swedish has two lexically specified tones, “accent 1” and “accent 2” (these are often labeled in scientific texts by superscript numbers before the syllable associated with the tone, e.g.,^1^ *munnen*, 2 *munnar*). Although the tones are realized on the stressed syllable of the word stem, their specification is based overwhelmingly on grammatical morphemes (Riad, 2014). Thus, accent 2 in ^2^ *munn‐ar*, mouth‐pl, “mouths” is realized on the stem *mun(n)*, but related to the plural suffix *‐ar*. In isolation, ^1^ *mun,* “mouth” carries accent 1 (Riad, 2014; Rischel, 1963). Thus, many suffixes induce^1^ a tone change to accent 2, that is, they lead to accent 2 being realized on the stem. Other suffixes are associated with accent 1 like the definite singular suffix *‐en*, “the” in ^1^ *munn‐en*, “the mouth.” Since Swedish tones are chiefly specified for grammatical morphemes, their lexical function is marginal (Elert, 1972). The majority of the few existing tonal minimal pairs emerge in inflected words due to homonymous suffixes that differ from each other concerning tone assignment (e.g., ^1^ *gift‐er,* marry‐prs, “marry/marries” vs ^2^ *gift‐er,* poison‐pl, “poisons” or ^1^ *håll‐et*, direction‐def, “the direction” vs ^2*håll‐*^ *et*, hold‐pst.ptcp.sg, “held”; Elert, 1972). Importantly, the strong interaction with suffixes allows native listeners to use the tonal information on word stems to pre‐activate possible upcoming word endings (Roll, 2015; Roll et al., 2013; Söderström et al., 2017). In consequence, tones in Swedish have been argued to be critical in the rapid differentiation of, for instance, singular and plural nouns in natural language comprehension.

**FIGURE 1:**
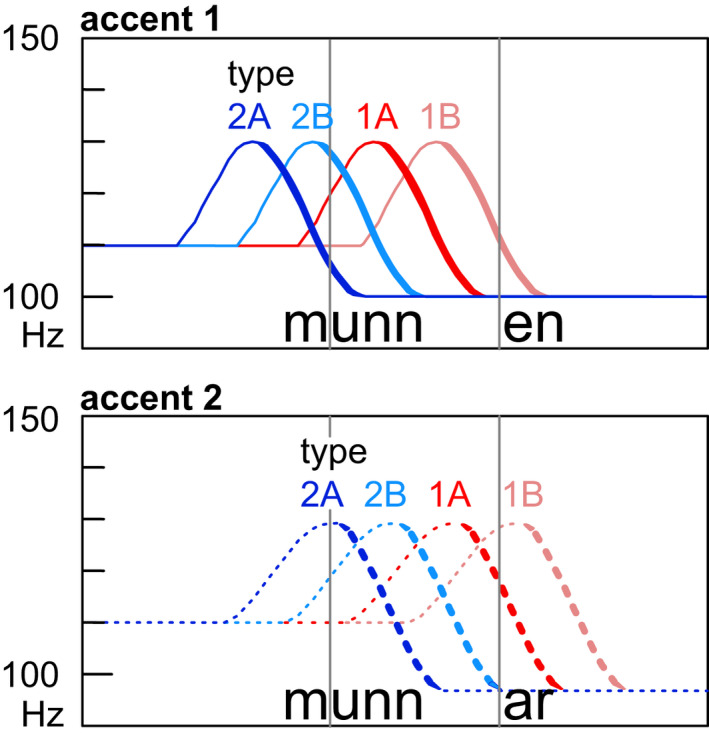
Stylized word accent patterns for a nonfocal realization of an accent 1 word (^1^ *munn‐en*, “the mouth,” above) and an accent 2 word (^2^ *munn‐ar*, “mouths,” below) in the four large dialect types in Sweden (2A = Central Swedish, 2B = West Swedish, 1A = South Swedish, 1B = Dalarna Swedish). Vertical gray lines indicate vowel onset. Adapted from Bruce (1983)

Regarding the phonetic pitch shape of the Swedish tones, both accent 1 and accent 2 are characterized by a falling pitch contour (e.g., Bruce, 1977, 1983, 2005; Riad, 2014). Interestingly, the onset of the fall is earlier for accent 1 than for accent 2, although the exact timing differs between dialects (Figure 1). Central Swedish, the standard variety (type 2A), has the overall earliest pitch fall timing: it is so early that accent 1 is realized as a low tone on the word stem and the preceding high tone becomes associated with the pretonic syllable. The word accents interact to some degree with sentence‐level prosody, such as focus or boundary tones. Focus, for instance, produces an additional rise following the word accent fall in some dialects (type 2 in Figure 1) while it increases the range of the word accent fall in other dialects (type 1 in Figure 1).

### 1.2 Tone and second language learning

The importance of tone in many languages in the world almost automatically entails a large number of people who acquire tone as part of a second language. In fact, for Mandarin alone, there are an estimated 200 million L2 speakers; and for Hausa, the largest African tone language, 25 million people are assumed to speak it as an L2 (Eberhard et al., 2020). Learning a language with nonnative tone is challenging, particularly for nontonal L1 speakers. Difficulties arise not only in L2 tone production but notably also in tone perception. Problems in this context range from basic phonetic tone discrimination and identification abilities to the phonological, categorical use of tone necessary for the distinction of lexical items and grammatical features. The process is likely hierarchical such that phonetic discrimination abilities need to be in place before phonological tone categories can be established and subsequently functionalized to identify and acquire tonally distinguished words or grammatical features (Wong & Perrachione, 2007). We will illustrate below how previous studies, predominantly on lexical tone systems in Asia, have strengthened this claim.

#### 1.2.1 Behavioral indices of L2 tone acquisition

The majority of previous studies on tone acquisition have investigated behavioral correlations of tone identification or tone discrimination abilities in L2 tone learners. They have typically found that advanced learners can reach fairly high identification accuracies but still perform below native speakers, at least for some tones (Gottfried & Suiter, 1997; Pelzl et al., 2019). Importantly, successful tone identification ability predicts learners’ ability to distinguish words at the lexical level (Ling & Grüter, 2020), but lexical recognition remains challenging even for learners who can confidently distinguish and identify tones (Pelzl et al., 2019). This is further complicated by phonetic variation within the phonological categories for tones, caused, for instance, by combinatorial constraints in nonmonosyllabic words (Chang & Bowles, 2015; Pelzl et al., 2019). Thus, as Wong and Perrachione (2007) suggest, low‐level phonetic and phonological knowledge appears to be a requirement for the use of tones for lexical decisions. Interestingly, it has also been shown that once learners have made an association between a specific segmental and suprasegmental unit, they find it easier to re‐access this particular association for further learning (Liu & Wiener, 2020). This shows that learners do not rely solely on phonological categories but also on previously learned associations.

Liu and Wiener (2020)’s finding closely relates to previous studies, suggesting that L2 tone perception and, in turn, the acquisition of tonal words is largely moulded by previous language experience. In this context, it was shown that speakers of contour tone languages differentiate foreign tone contrasts with the help of pitch cues pertaining to both tone height and, significantly, tone movement direction (Gandour, 1983). Speakers of nontonal native languages (and likely speakers of register tone languages), in comparison, predominantly classify tone contrasts with respect to pitch height (Gandour, 1983; Huang & Johnson, 2010), as tone directionality has no word‐ or syllable‐level relevance in their L1. This lack of learned attentional focus on tone movement often results in decreased perception proficiency for L2 tone contours for nontonal learners (Burnham et al., 2015; Liu, 2013; Qin & Mok, 2011; Yu et al., 2019). While familiarity with tone movement and tone height are good general indicators of L2 tone processing, it is likely that the actual influence of L1 or previously learned tone is considerably more fine‐grained. It has been shown that general attention to movement and specific native phonological categories often guide L2 tone perception and categorization Chen et al., 2020; So & Best, 2014). Tonal L1 speakers’ knowledge of tone patterning may even lead to their outperforming speakers of nontonal languages at automatic tracking of the L2s tonal phonotactics (Chan & Leung, 2020).

#### 1.2.2 Electrophysiological indices of L2 tone acquisition

The acquisition of L2 tone can also be studied with the help of electrophysiological measures. Measuring the electric voltage on participants’ scalp while they listen to L2 tones can inform on how their brains process the incoming information. There are different listening paradigms and different electrophysiological responses that are relevant in this respect.

The earliest known language‐related electrophysiological response is a very early component that emerges in the neural activity around 50 ms after the stimulus divergence point (DP, i.e., the point in time when the stimulus becomes physically different from other stimuli with similar onsets). The component at 50 ms post‐stimulus has not yet received a uniform label but is characterized by being sensitive to lexicosemantic (MacGregor et al., 2012; Shtyrov & Lenzen, 2017) and syntactic (Herrmann, Maess, & Friederici, 2011) properties of spoken words. Particularly, it is argued to be related to the automatic assessment of words’ linguistic properties, such as lexicality status or syntactic category, suggesting it reflects a pre‐attentive gating response. Although relatively novel, the effect is stable. It has been observed in many different languages (English: Shtyrov & Lenzen, 2017, Finnish: Kimppa et al., 2015, German: Herrmann et al., 2009, Danish: Partanen et al., 2017, Chinese: Yue et al., 2014), using varying paradigms (ignore conditions: Shtyrov & Lenzen, 2017, attend conditions: Kimppa et al., 2015, tasks: Herrmann, Maess, & Friederici, 2011, oddball paradigms: MacGregor et al., 2015, single word presentations: Partanen et al., 2017, sentence presentations: Herrmann, Maess, Hahne, et al., 2011, or acquisition contexts: Gosselke Berthelsen et al., 2020) and different listener populations (healthy adults: MacGregor et al., 2012, children: Partanen et al., 2017, aphasics: MacGregor et al., 2015, or L2 learners: Kimppa et al., 2019). For language learning, the component distinguishes newly learned words from nonwords within just minutes of word acquisition/exposure. It can, therefore, serve as an indicator of memory trace formation. The component also seems well‐suited for studying tone word processing, as previously illustrated by Yue et al. (2014). They played highly frequent Mandarin word forms (i.e., *tang3*, “to lie down,” “to drip,” “if,” and *peng3*, “to praise,” “to offer,” “to clasp”) and very infrequent or nonexistent word forms (i.e., *teng3*, a pseudoword, and *pang3*, “to weed,” a rare word incorrectly introduced by Yue et al. as a pseudoword) to native listeners in a passive listening paradigm. They found an initially reduced negativity to infrequently presented uncommon and nonexistent words compared with frequently presented real words that quickly (i.e., within minutes) increased and became a comparatively larger negativity. The same activation pattern has been observed for novel nontonal word forms (Kimppa et al., 2015). It is believed to signal enhanced activation reflecting an ongoing process of memory trace formation. This lexicality gating response has not, however, been extensively studied for L2 learning. Yet, Gosselke Berthelsen et al. (2020) found increased neural activity for pseudowords—or decreased activity for meaningful novel words—in learners with a tonal native language, suggesting an impact of language experience in this early component. However, more research into the effect is needed for both L1 and L2 tone processing.

A second electrophysiological response worth mentioning is the mismatch negativity (MMN; Näätänen et al., 1978). The MMN is an automatic, pre‐attentive brain response that occurs before listeners are consciously aware of hearing a stimulus and even when they pay no attention to the auditory input (Näätänen & Alho, 1995). The response indexes whether the listeners’ brain can detect a change in the input stimuli. Specifically, in an oddball paradigm, participants listen to many repetitions of a standard stimulus intermixed with rare occurrences of deviant stimuli. Researchers then study the neural responses to see if the difference between standards and deviants has been detected. If this is the case, a stronger MMN response is elicited for the deviant. As regards tone processing, Shen and Froud (2019) found a mismatch negativity for phonemic but not phonetic tone differences for native speakers. For nontonal learners and nonlearners, in comparison, only pitch intervals but not phonological categories influenced the MMN (Chandrasekaran et al., 2007; Yu et al., 2019). Further, MMNs were reduced at large stimulus intervals (Yu et al., 2017). Interestingly, learners with a tonal L1 showed a mixed response. That is, their MMNs varied as a function of both phonemic differences and pitch intervals. This reinforces the behavioral results and suggests a relatively strong influence of the learners’ L1 both on behavioral responses and at pre‐attentive tone processing stages. Interestingly, Shen and Froud (2019) showed that the influence of L1‐shaped perception is retained, albeit to a lower degree, even in relatively advanced learners.

Finally, also relevant in the context of tone processing is a relatively late ERP deflection expressed as N400 or AN (anterior negativity). At a response latency of around 400 ms after the presentation of the stimulus, the N400 is sensitive to semantics while the AN (also LAN, left anterior negativity, since it is often left‐lateralized) is indicative of grammar processing (decomposition). The N400 is often attenuated outside the focus of attention (McCarthy & Nobre, 1993; Okita & Jibu, 1998), which suggests that responses at this latency are dependent on attention allocation to speech input unlike the very early component at ~50 ms or the fully pre‐attentive MMN at ~150 ms. The N400 and AN components are elicited naturally for any attended linguistic input, which makes it possible to investigate how they are affected by different linguistic factors (Blomberg et al., 2020; Krott & Lebib, 2013). Most often, however, both of these responses are investigated in the context of violations, since they are amplified for incongruent or incorrect input. The increase in the N400 or AN due to incongruent/incorrect language is what we will in the following refer to as an “N400 effect” and “AN effect,” respectively (Kutas & Federmeier, 2011; Kutas & Hillyard, 1980; Osterhout & Mobley, 1995; Rodriguez‐Fornells et al., 2001; Schremm et al., 2019). N400 and AN effects have also been observed in the context of tone processing. In languages where tone has a lexical function, changing the tone on a target word changes its lexicosemantic content and thus turns it into a bad fit for the context. Such tone mismatches evoke N400 effects in native speakers (Brown‐Schmidt & Canseco‐Gonzalez, 2004; Ho et al., 2019; Malins & Joanisse, 2012; Pelzl et al., 2019; Zhao et al., 2011). In a language where tone has strong associations with following grammatical suffixes, like Swedish, an anterior negativity has been found for tone‐suffix mismatches when there is maximal focus on rule‐based processing and the grammatical content (Söderström et al., 2017).

A number of L2 studies have found only limited late N400 or AN effects for L2 tone errors, in particular for nontonal L1 learners. In a study involving learning a language with grammar‐associated tone, beginner and intermediate learners from a nontonal L1 showed neither an N400 or AN effect before intensive training (Gosselke Berthelsen et al., 2018; Hed et al., 2019). Similarly, in a study on advanced nontonal L1 learners of a lexical tone language, there was no group‐level N400 effect after tone mismatches, although pitch discrimination abilities were high; however, individual learners did show an N400 effect (Pelzl et al., 2021). An N400 effect has also been found for learners with an intensive training paradigm with a limited number of tonal words (Dittinger et al., 2016). Directly comparing tonal and nontonal beginner learners’ acquisition of words with grammatical L2 tone, it has been found that tone‐picture mismatches evoked an N400 effect^2^ only in learners with a tonal L1 (Gosselke Berthelsen et al., 2021).

While still relatively sparse, the above‐described neurophysiological findings for tone processing support the idea that tone acquisition builds incrementally on phonetic and phonological knowledge. Basic phonetic tone discrimination skills precede phonological tone categorization, as suggested by the MMN results. Pure pitch‐based discrimination is possible to some degree, even for nonlearners. Only relatively advanced learners with a tonal L1, on the other hand, show MMNs that are influenced by the L2’s tonal categories. Finally, tone‐meaning associations and lexical and grammatical learning, visible in N400 and AN effects, occur only at very advanced stages of learning or after intensive perceptual and associative training. The presented electrophysiological results also stress the beneficial effect of having L1 tone experience in L2 tone processing. It has been assumed, in this context, that tonal information storage and processing is underpinned by the left planum temporale for L1 speakers of a tone language (Schremm et al., 2018). This might be a prerequisite for rapid, more native‐like processing of foreign tones. With intensive tone‐focused training or at high L2 proficiency, however, learners with a nontonal L1 might be able to overcome native‐language biases and produce tone‐mismatch‐related ERP responses for L2 tone (Dittinger et al., 2016; Hed et al., 2019; Pelzl et al., 2021).

### 1.3 The present study

The above‐outlined literature has provided important insights into the processing of L2 tone. Importantly, it has shown a reliance on phonetic knowledge for higher level tone learning to occur, such as the mapping between tone and lexical or grammatical content. It is likely that the acquisition of grammatical tone is easier than that of lexical tone as grammatical function is mapped directly onto the phonological tone categories rather than the tones being meaningful in association with segmental information only. Due to their previous phonetic sensitivity and phonological experience with tones, lexical tone speakers may be able to acquire grammatical tone more easily than grammatical tone speakers do lexical tone. In general, previous research has suggested that L1 familiarity with tone or other previous tone experience can facilitate the discrimination of L2 tone or the acquisition of novel tonal words. However, it is largely unknown what degree of familiarity or similarity is needed for facilitation to occur, and if transfer facilitates grammatical tone learning, too. It is also unclear whether L1–L2‐based facilitation affects all levels of tone processing alike.

To address some of these issues, we studied two groups of learners during their acquisition of artificial novel words with grammatical tone. All learners came from closely related and highly similar languages, but one group’s native language was tonal (Swedish), and the other’s was not (German). We manipulated the similarity of L1 and L2 tone for the tonal learners (contour tones [fall, rise] vs level tones [high, low]) and investigated different types of learning responses: the pre‐attentive gating response for auditory stimuli, late responses related to lexical and grammatical processing, and behavioral responses for mismatch detection. Further, because we chose a grammatical type of tone, tones could be studied independently of the lexical items they were attached to. This also allows us to investigate the importance of the learners’ familiarity with the tone’s linguistic function directly—in this case, the expression of grammatical meaning. It is possible, for instance, that we find amplitude differences in the N400/AN for all novel words with native‐like tone patterns in the early acquisition stages as a form of direct transfer and L2 processing through the lens of the L1 (e.g., a reduced N400 for easier lexicosemantic processing compared with pseudowords or an increased AN for rule‐based processing after successful rule acquisition). In the behavioral data, investigating tone mismatch identification, we could study the behavioral correlates of tone acquisition fully isolated from the segmental information. Any familiarity‐based advantage observed at this point could be directly attributed to L1–L2 familiarity. As the grammatical tones that we used involved a one‐to‐one mapping with meaning, it is likely that phonetic attunement and categorization into phonologically relevant units would directly and immediately open up for the acquisition of the tone’s grammatical content. We assumed that if grammatical function and phonological similarity of L1 and L2 tones would be the determining factor in L2 tone acquisition, the tonal speakers’ learned attention to pitch movement should result in facilitated tone‐grammar learning (faster word trace formation and changes in N400/AN^3^) for words with tonal movements, possibly even restricted to falling pitch, as the only tone that has word‐level relevance in their L1. If, however, general experience with grammatical tone is sufficient to facilitate L2 tone acquisition, we should see no differences between tone types for the tonal L1 speakers but still clear differences between tonal and nontonal learners. If L1 experience should not influence the discrimination of tones and the mapping between tone and grammar in its initial stages, there should be no processing differences between learners from different L1 backgrounds. Finally, the different responses that we investigated could be affected differently by L1–L2 familiarity. It has previously been shown that transfer affects early processing stages more than behavioral responses and late processing stages (Andersson et al., 2019). Therefore, we expected the strongest effect of familiarity in the early response.

## 2 METHOD

### 2.1 Participants

Forty‐eight healthy, right‐handed adults (mean age 23.7, 25 females) with normal or corrected‐to‐normal vision and normal hearing (defined as pure‐tone hearing thresholds ≤20 dB Hearing Level (ISO, 2004) were recruited for the study. All tests were carried out at the Lund University Humanities Lab, and most participants were students at Lund University. Half of the participants had a tonal L1, Swedish, the other half a nontonal L1, German. Twenty‐four participants were chosen per group as to allow for counterbalancing of vowels and tones between groups; see below.

The tonal and nontonal participants were each divided into two learner groups: high/fall learners (i.e., participants who were taught target words with high and falling tones, where low and rising tones served as controls) and low/rise learners (i.e., participants who were taught target words with low and rising tones, where high and falling tones served as controls). The division into high/fall vs low/rise was based on the desired property of all target words to initially have identical pitch and be indistinguishable from each before the onset of the vowel, which was also the onset of the tone movement. In this way, we obtained a clear divergence point for the ERP data. All four groups (i.e., tonal L1 high/fall, tonal L1 low/rise, nontonal L1 high/fall, nontonal L1 low/rise) were matched for age (23‐24 years), gender (6 females per group), socioeconomic status (Hollingshead, 1975), working memory span (Unsworth et al., 2005), their perception of nontonal phonological contrasts (i.e., vowel duration: mean accuracy 97.1%) and their discrimination of extra‐linguistic pitch (i.e., piano tones: mean accuracy 92.5%). All subjects were remunerated for their participation. One tonal L1 high/fall participant reported previous exposure to a foreign tone language and one participant from the nontonal L1 low/rise group chose to discontinue the experiment. The data from both participants were excluded. The experiment was carried out according to the guidelines in the Declaration of Helsinki and approved by the local ethics review board in Lund.

### 2.2 Stimuli

**FIGURE 2:**
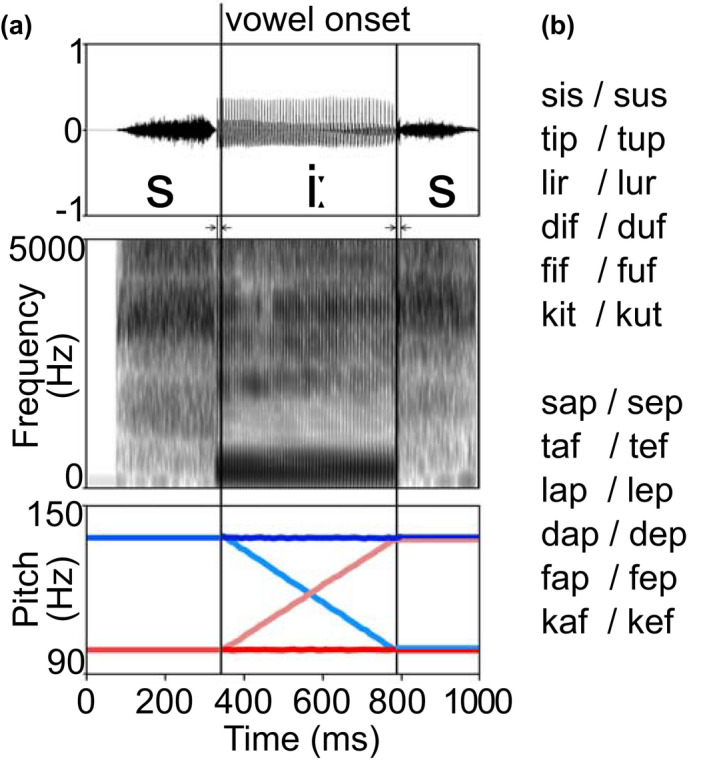
(a) Example of an auditory stimulus with waveform and spectrogram. The four possible pitch patterns are indicated in colors below. Half of the participants learned the red patterns (low/rise), the other half, the blue patterns (high/fall). (b) Full list of auditory stimuli used in the experiment

To test tone processing and tone word acquisition, we created 24 tonal pseudowords. We chose an artificial language instead of a natural grammatical tone language in order to most strongly control the experimental situation. Artificial language learning has been shown to correlate strongly with natural language learning and was, therefore, deemed suitable for this experiment (cf. Ettlinger et al., 2016). All test words had a simple CVC structure (consonant‐vowel‐consonant), for instance /siːs/, a frequent structure for monosyllables in German and English. Monosyllables were deemed suitable for studying transfer from Swedish as the domain of tone in Swedish is the word rather than the syllable (in contrast to, e.g., Mandarin) and because monosyllabic stems frequently and most consistently undergo tone changes. All consonants and vowels were recorded separately in an anechoic chamber by a male speaker of Russian (to prevent a bias that would arise should the speaker come from one of the two experimental L1s). During the recording, consonants were preceded or followed by two dummy vowels (/o/, /ø/) for their naturalistic pronunciation unconfounded by coarticulation with some of the actual stimuli’s vowels. These dummy vowels were cut off before splicing the consonants with the actual vowels used for the main test words (/a/, /ε/, /i/, /u/). The initial consonants, vowels, and final consonants were then equalized for length and loudness and spliced together in Praat (Boersma, 2001) with 10 ms transition phases. All resulting pseudowords were 1000 ms long (C = 328 ms, V = 464 ms, C = 218 ms) with short silent closures before an initial and after a final plosive. The employed Russian phonemes, while similar to both German and Swedish ones, were chosen to avoid differential carry‐over effects which German or Swedish phonemes could have evoked. All test words were perceived equally well and correctly classified as pseudowords by eight German speakers and three Swedish speakers who were not participants in the main study. In a final step, we used pitch manipulations to add two level tones (high: 138 Hz and low: 98 Hz) and two contour tones, a rise (98 Hz to 138 Hz) and a fall (138 Hz to 98 Hz). The pitch was selected in accordance with the speaker’s natural pitch range. The pitch movements had a naturalistic pitch span (40 Hz, 6 semitones) compared with the pitch movements of Swedish word tones and were easily distinguished by several native speakers of German and Swedish who piloted the experiment. The onset of pitch movement was aligned with the onset of the vowel (cf. Figure 2) in order to define a single earliest possible point at which the stimuli could be identified. This point was used as a time‐locking point for the ERP data. Yet, while it is the earliest point at which the stimuli diverge, it would take a few milliseconds for the listeners to distinguish and correctly identify the stimuli. This presumably varied slightly, both intra‐ and interpersonally.

**FIGURE 3:**
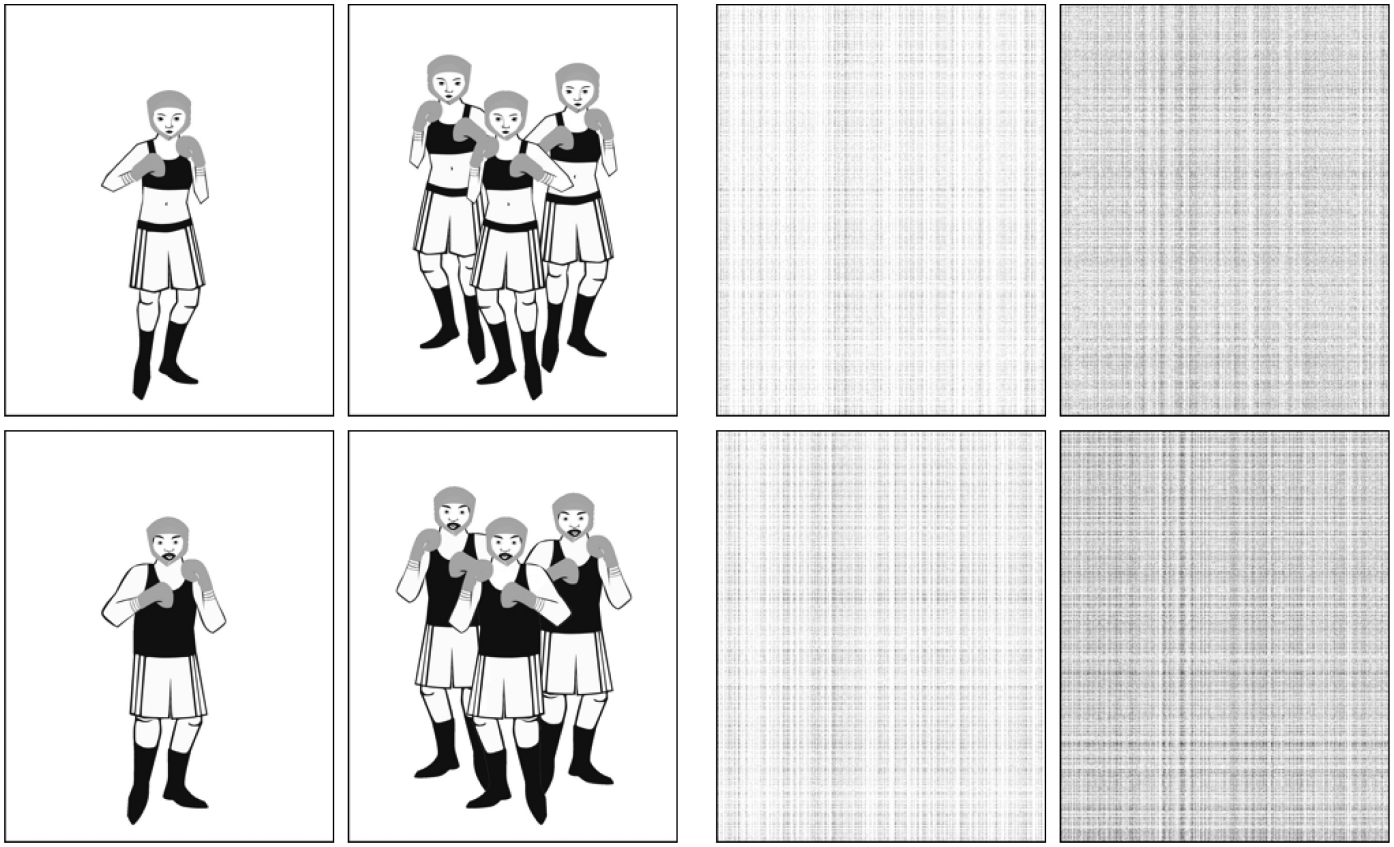
A set of picture stimuli depicting singular and plural versions of male and female boxers and the corresponding control pictures. Control pictures were presented pseudorandomly with different control words such that there were no meaningful patterns

For the stimuli to become meaningful, we taught them through association with meaningful pictures. The pictures which we constructed for this purpose showed people in 24 different professions. For each profession, versions of the pictures illustrating gender (i.e., masculine = male worker, feminine = female worker) and number (i.e., singular = one worker, plural = two, three, or four workers) were created (cf. Figure 3). We used inflectional categories that were easily expressed through pictures in order to avoid a situation where participants relied on their native language or a common third language to disambiguate the new words (which could have led to unwanted transfer effects). Both German and Swedish participants are familiar with gender and number inflections on nouns that designate professions: e.g., German *Lehrer‐inn‐en*, teacher‐fem‐pl, “female teachers” or Swedish *lärar‐inn‐or*, teacher‐fem ‐pl, “female teachers.” While the use of traditional gender suffixes has become less frequent in Swedish, alternative gender‐specifying suffixes like *‐kvinna, “‐*woman,” have gained popularity (e.g., *tales‐kvinna*, “spokes‐woman” rather than *tales‐man*, “spokes‐man”; cf. Hornscheidt, 2003). Thus, both German and Swedish participants are familiar with inflections that express gender and number in job titles. An essential difference between the groups is that, in Swedish, inflections can induce a tone change on the preceding word stem. Thus, tone can be seen as a leftward extension of, for instance, singular/plural suffixes (cf., ^1^ *smed*, smith.sg, ^2^ *smed‐er*, smith‐pl). In contrast to number, gender in Swedish is not supported by tonal differences, but we hypothesized that the mere presence of solid associations between tone and inflections in Swedish would make its speakers susceptible to any such contrast in a second language. In German, tone is not a lexical or grammatical feature and is not associated with the inflectional system, and the German participants were, thus, unfamiliar with the use of tones for the emphasis or expression of inflectional categories. Before including the pictures in the study, we tested them on a number of German and Swedish native speakers to ensure that the intended grammatical meaning was easily perceived. For the main study, we explicitly demonstrated how number and gender would be expressed by including explicit instructions with animal pictures (lion‐ess‐es) and a short training paradigm with simple Spanish words (*arquitecto/a‐s*, architect.mas/fem‐pl). Debriefing with the participants after the study confirmed that they had all correctly identified the intended meanings of the pictures in the main study.

In addition to the meaning‐assigning profession pictures, nonmeaningful pictures (with scrambled gray vertical and horizontal patches, matching the meaningful images in black/white pixel balance) were added for use with the nontaught control words for balancing the conditions for their basic visual properties.

### 2.3 Procedure

**FIGURE 4:**
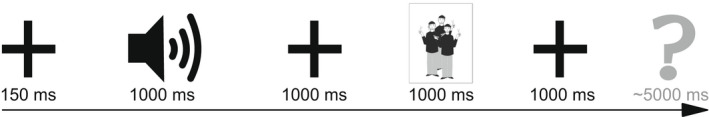
Experiment procedure. The question mark in gray illustrates the addition of a question to 12% of all trials. In question trials, a question concerning the correctness of the previous word‐picture pair was followed by overt feedback and a repetition of the auditory stimulus and the associated visual stimulus

Each artificial L2 learner participated and learned 24 words on two consecutive days. At their arrival, participants gave written consent about participating in the study. During EEG application on the first day, they filled in a number of background questionnaires. They were subsequently seated at a fixed distance from a computer screen on which the visual stimuli, fixation crosses, questions, and feedback were presented. These were created in and controlled by E‐Prime 2 stimulation software (Psychology Software Tools Inc., Sharpsburg, PA). All subjects were asked to keep their index fingers on a response box on a table in front of them to answer questions when prompted. The auditory stimuli in the experiment were routed through a GSI 16 Audiometer (Grason & Stadler Inc., Eden Prairie, MN) and presented at 70 dB SPL through a pair of circumaural earphones (California Headphone Company, Danville, CA). The presentation level was verified using a sound level meter (Brüel and Kjær 2231, with a 4134 microphone in a 4153 Artificial Ear). At the very beginning of the first learning session, participants received explicit instructions regarding the learning paradigm and conducted a test phase which illustrated the learning procedure with the help of Spanish words with gender and number suffixes (i.e., *mechanico/a(s)*, mechanic.mas/fem(pl), “mechanic(s)” and *arquitecto/a(s)*, architect.mas/fem(pl), “architect(s)”). Participants then listened to all auditory stimuli once before the learning procedure. This pure auditory repetition was for basic familiarization purposes only and not included in the statistical analysis. Afterwards, the learning procedure commenced with an auditory stimulus followed by a meaning‐giving picture (cf. Figure 4). Stimulus onset asynchrony was 4.15 seconds. Meaning assignment was strongly regulated so that the initial consonant always assigned lexicosemantic information (i.e., the profession), while the vowel and the tone were associated with the grammatical categories gender and number. Across participants, we counterbalanced the distribution of professions as well as whether vowel or tone was associated with gender or number. Two vowels (12 stimuli per vowel) and two tones (12 stimuli per tone) were part of the learning paradigm (target words), while the other two tones and two vowels (12 stimuli each) were used in control words which were presented with nonmeaningful pictures. An example of a full set of auditory stimuli and their meaning for one participant can be found in Table 1.

**TABLE 1:** Example of a full set of stimuli for one participant. Tones are indicated according to the international phonetic association’s notation: ´ = high, ` = low, ^ = fall, ˇ = rise

Target words					Control words		
Vowel (gender)					Vowel		
Tone (number)	díf	*waiter.fem.pl*	dúf	*waiter.mas.pl*	Tone	dàp	dèp
	dîf	*waiter.fem.sg*	dûf	*waiter.mas.sg*		dǎp	děp
							
	fíf	*hairdresser.fem.pl*	fúf	*hairdresser.mas.pl*		fàp	fèp
	fîf	*hairdresser.fem.sg*	fûf	*hairdresser.mas.sg*		fǎp	fěp
							
	kít	*race driver.fem.pl*	kút	*race driver.mas.pl*		kàf	kèf
	kît	*race driver.fem.sg*	kût	*race driver.mas.sg*		kǎf	kěf
							
	lír	*flautist.fem.pl*	lúr	*flautist.mas.pl*		làp	lèp
	lîr	*flautist.fem.sg*	lûr	*flautist.mas.sg*		lǎp	lěp
							
	sís	*boxer.fem.pl*	sús	*boxer.mas.pl*		sàp	sèp
	sîs	*boxer.fem.sg*	sûs	*boxer.mas.sg*		sǎp	sěp
							
	típ	*cook.fem.pl*	túp	*cook.mas.pl*		tàf	tèf
	tîp	*cook.fem.sg*	tûp	*cook.mas.sg*		tǎf	těf

*Notes*: Target words are followed by their intended meaning for this participant in italics. Vowel and tone differences are related to grammatical properties. Note that consonants encode profession.

Control words served as comparison stimuli to distinguish learning from familiarization. For vowels, a/ε or i/u were paired together. Each pair was equally often included as part of the target or control words. Tones were split up in the same way, ensuring that word onsets for the target words for each participant had identical pitch levels. This resulted in a high/fall group and a low/rise group. In the high/fall group, target words had high or falling pitch, while the controls had low and rising tones. For the low/rise group, the pattern was inversed. Finally, to allow behavioral assessment of the learning progress, the stimulus and the subsequent picture were mismatched in approximately 6 percent of all trials. Together with 6 percent of the congruous trials, these served as catch question trials. In a question trial, participants were asked to judge the correctness of the previous word‐picture pair. When participants answered with the help of the button box, overt feedback was provided. Depending on response times, question trials were approximately 5 seconds longer than nonquestion trials. On each day, every stimulus (24 targets and 24 controls) was repeated 30 times in nonquestion trials and at least once in a question trial (30 in total). Thus, 2880 trials per participant were analyzed for ERP effects (48 trials × 30 repetitions × 2 days). They consisted of 1440 target trials (720 contour tones, 720 level tones) and 1440 control trials (720 contour tones, 720 level tones). About 180 of the target trials were question trials. Together with an additional 180 mismatch trials, these formed the basis for the behavioral data. For analysis, the stimuli were grouped into blocks of five repetitions. Participants were offered a longer break after every ten repetitions (~40 min).

### 2.4 Electrophysiology

During the acquisition paradigm, the participants’ brain activity to the (nonviolated) auditory stimuli was recorded with the help of 64 Ag‐AgCl EEG electrodes mounted in an electrode cap (EASYCAP GmbH, Herrsching, Germany), a SynAmps^2^ EEG amplifier (Compumedics Neuroscan, Victoria, Australia), and Curry Neuroimaging Suite 7 software (Compumedics Neuroscan). We monitored eye movements with horizontal and vertical bipolar electrooculogram electrodes (EOG). The impedance at scalp channels was kept below 3 kΩ and below 10 kΩ for the eye channels. Online reference was left mastoid (M1), and frontocentral electrode AFz served as ground. We recorded EEG with a 500 Hz sampling rate using DC mode and an online anti‐aliasing low‐pass filter at 200 Hz. We subsequently filtered the data with a 0.01 Hz high‐pass and a 30 Hz low‐pass filter. About 1200‐ms long ERP epochs (including a 200‐ms baseline) were extracted, time‐locked to the divergence point. Vowel onset was chosen as the ERP time‐locking point as it is the earliest point at which the stimuli physically diverged; although the point at which individual participants would identify the stimuli could differ slightly from vowel/tone onset, our choice of time‐locking point was in keeping with the bulk of previous ERP studies that used physical divergence as an objective criterion for aligning brain responses to. An independent component analysis (ICA; Jung et al., 2000) was conducted and components related to eye artefacts and bad channels were removed. Epochs still exceeding ± 100 μV were discarded. The ICA components were well defined due to a high number of trials in the paradigm that we could submit to ICA analysis (3156 auditory stimuli per participant = 96 familiarization trials [before learning], 2880 learning trials [targets and controls], 180 mismatch trials [which were congruent during the auditory stimuli]). Consequentially, the rejection of, on average, 5 out of 66 ICA components was sufficient to correct the data to such a degree that only a few trials (*M* = 35 per participant) exceeded the 100 μV threshold. While familiarization and mismatch trials were included during ICA, only learning trials were submitted to statistical analysis.

### 2.5 Statistical analysis

#### 2.5.1 Behavioral data

To test for possible effects of L1–L2 similarity in the behavioral results, we analyzed the behavioral responses to tone mismatch trials focusing on differences between words with contour tones and words with level tones. To this effect, we separately submitted mean data for the behavioral variables “Response Accuracy” and “Response Times” to two mixed analyses of variance (ANOVA) with the experimental factor “Tone Type” (contour tones vs level tones), the temporal factor “Day” (day 1 vs day 2) and the between‐subject factors “Learner Group” (tonal L1s vs nontonal L1s) and “Target Tone Group” (high/fall vs low/rise). Response Times were normalized through log transformations, and for accuracy, d′ scores were computed. As two participants were excluded from the study, the Target Tone subgroups were of different sizes (11 or 12). We used mean imputation wherever same‐sized target groups were necessary for the statistical analysis. All behavioral analyses were carried out in SPSS 26 (International Business Machines [IBM] Corp., Armonk, NY, United States).

#### 2.5.2 ERPs

For the ERP data, we selected two time windows (50–70 ms and 400–600 ms) where we expected to observe influences of L1–L2 similarity on tonal or tone features on word processing, based on previous literature (Gosselke Berthelsen et al., 2018, 2020). Using these pre‐defined time windows, we conducted cluster‐based permutation tests for the factor “Tone Type.” We submitted mean ERP amplitudes of block 1 (on day 1) from both participant groups, together and separately, for the selected time windows and conditions (i.e., words with contour tones compared to words with level tones) to a permutation analysis using the nonparametric cluster‐based permutation approach implemented in Fieldtrip toolbox for Matlab (Maris & Oostenveld, 2007). We ran 1000 random permutations of the data with the Monte–Carlo method to account for large data sets and considered clusters of three or more electrodes with a *p*‐value of <.05 significant. We additionally tested for interactions with “Learning” (target word vs control word) in the permutation analysis to see whether target words differed from control words. The interaction was particularly important for the word recognition effect at 50 ms, where tonal learners have earlier been seen to automatically dissociate target words from control words (Gosselke Berthelsen et al., 2020).

If significant clusters emerged in any analyses, we carried out mixed ANOVAs to test for possible interactions with temporal and between‐subject factors. Thus, we computed one mean ERP amplitude across the analyzed time windows and all cluster electrodes. This was done for each participant in each block and day. The thus obtained mean amplitudes were then submitted to a mixed ANOVA with the experimental within‐subject factors “Tone Type” and “Learning,” the temporal factors “Day” and “Block,” as well as the between‐subject factors “Learner Group” (if applicable) and “Tone Target Group.” The ANOVAs were carried out in SPSS 26 (IBM). Greenhouse‐Geisser correction was used where necessary. For multiple pairwise comparisons, False Discovery Rate (FDR) corrections (Benjamini & Hochberg, 1995) were applied.

#### 2.5.3 Correlations

To test whether ERP effects were affected by individual learning behavior, we carried out two two‐tailed Pearson correlations with the variables “Amplitude Change for Lexicality Effect” and “Amplitude Change for Anterior Negativity,” on the one hand, and “Response Time Change” and “Response Accuracy Change,” on the other. The change investigated here was defined as the difference in behavioral or ERP responses between the first and the second half of day 1, where the bulk of learning took place (cf. Gosselke Berthelsen et al., 2020). Correlation analyses were carried out in SPSS (IBM). FDR corrections were applied to the correlations’ p‐values.

## 3 RESULTS

### 3.1 Behavioral results

**FIGURE 5:**
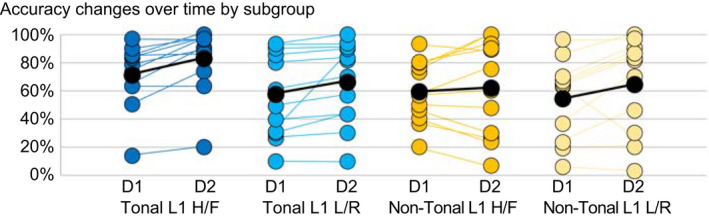
Accuracy changes over time by subgroup: Distribution of participants’ accuracy on tone error detection and accuracy changes between day 1 (D1) and day 2 (D2) by subgroup. Colored lines connect individual participants. Mean accuracy values per group and day indicated with black dots and mean accuracy change by black lines

For Response Accuracy, there was no significant main effect of the factor Tone Type and no significant interactions with the factors Tone Type, Learner Group, or Tone Target Group. A main effect of the temporal factor Day, *F*(1,44) = 44.18, *p* < .001, $\eta_{p}^{2}$ = 0.501, showed evidence of learning regardless of which sets of tones the learners acquired or which tone types were tested. Thus, Response Accuracy increased significantly from day 1 (*M* = 60.5%, *SD* = 25.4, range = 5.6 – 97.1) to day 2 (*M* = 68.8%, *SD* = 29.6, range = 2.9 – 100). For the descriptive statistics, we use percentages for the sake of simplicity and comparability across results and with other studies, whereas the statistical analysis was carried out on d′ data. For a graph illustrating the accuracy results and the change over time, please refer to Figure 5.

For Response Times, a significant interaction of Tone Type and Tone Target Group, *F*(1,44) = 4.63, *p* = .037, $\eta_{p}^{2}$ = 0.10, broke down into a main effect of Tone Type in the low/rise group, *F*(1,22) = 6.69, *p* = .015, $\eta_{p}^{2}$ = 0.23. Mismatches with the pictorial referent based on low tones (*M* = 1515 ms, *SD* = 986, range = 219 – 4279) were significantly faster detected than errors based on rising tones (*M* = 1722 ms, *SD* = 1093, range = 303 – 4241). There was no main effect of the factor Tone Type and no significant interactions involving the factors Tone Type, Learner Group, or Tone Target Group. Main effect of the temporal factor Day, *F*(1,44) = 35.53, *p* < .001, $\eta_{p}^{2}$ = 0.45, showed evidence of learning regardless of which tone types the learners were taught or tested on. To this end, there was a significant improvement in Response Times from day 1 (*M* = 2056 ms, *SD* = 329, range = 488 – 4279) to day 2 (*M* = 1427 ms, *SD* = 153, range = 199 – 4241). The analysis was carried out on log‐transformed data; the actual raw Response Times are also reported for data description.

### 3.2 ERP results

#### 3.2.1 50–70 ms

**FIGURE 6:**
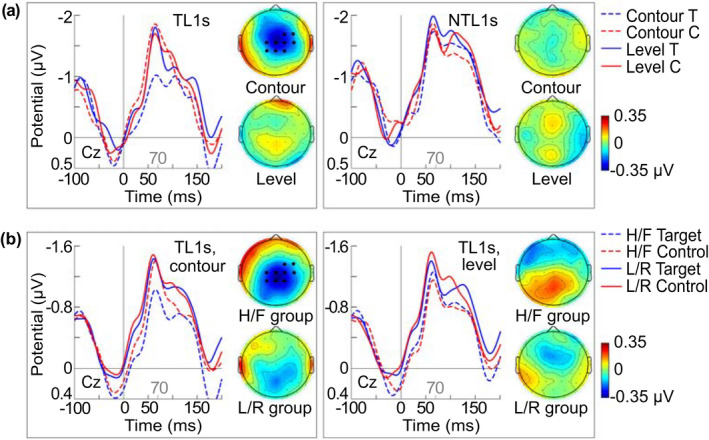
ERPs and subtraction topographies (controls minus targets) for the 50–70 ms effect. (a) ERPs for the first block of session 1 at central electrode Cz and topographies for the Tone Type by Learning interaction cluster of the permutation analysis (electrodes marked in black) in the tonal L1 (TL1) group (left) and comparable topographies for the nontonal L1 group (right). (b) ERPs for all trials on both days and topographies for the early effect in the tonal L1 group by Tone Type: Responses to contour tones (left) and level tones (right). T = Target words; C = Control words; H/F = high/fall group; L/R = low/rise group. For better visibility, only part of the epoch and the baseline are shown. More electrodes can be found in the Supporting Information S2a and Supporting Information S2b

**FIGURE 7:**
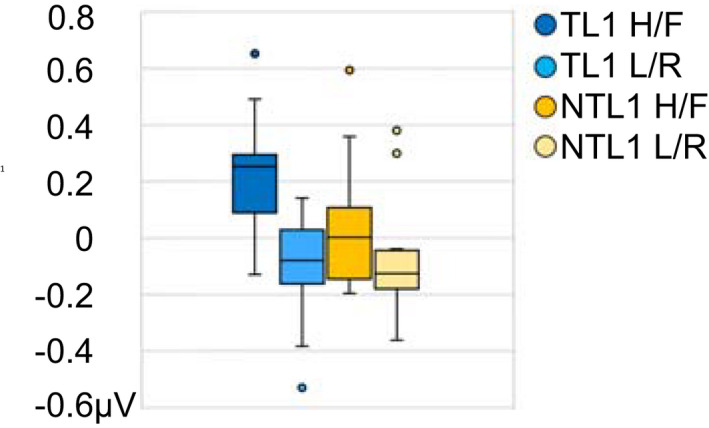
Box plot illustrating the distribution of the difference amplitude for the early ERP effect between pitch falls and pitch rises. Data shown by learner group and target tone subgroup, regardless of word status (i.e., targets or controls). The whiskers extend to the minimum and maximum data values within 1.5 times of the interquartile range. Outliers indicated by dots

For the early time window, an interaction between Tone Type and Learning, i.e., comparing level and contour tones in target and control words, produced a significant central electrode cluster (FC2, FC4, C1, Cz, C2, C4, CP1, Cpz, CP2), *p* = .026, *d* = 0.87, in the tonal L1 group. See ERPs and topographies for the interaction in Figure 6. No comparable cluster was identified in the nontonal L1 group or for all participants, collectively. The permutation analysis did not produce any significant clusters for differences between level and contour tones without an interaction with Learning (neither for all participants collectively nor for the participant groups separately). For a boxplot showing the subtraction amplitude distribution within and between groups, please see Figure 7.

Further investigating the significant cluster of the tonal L1 group in a mixed ANOVA, a Tone Type * Learning * Target Tone Group interaction suggested that the observed effect for contour tones (i.e., controls were more negative than targets) was significant in the high/fall group only. Secondly, an interaction with time indicated that only amplitudes of control words changed over time, turning less negative. For detailed results, see Table 2.

**TABLE 2:** All significant results of the mixed Analysis of Variance analysis (ANOVA) for the tonal L1 learners’ ERPs in the early time window

Effects and interactions	*F*	DF	*p*	$\eta_{p}^{2}$		Means in μV	Std Error	95% Confidence interval
Tone Type × Learning	9.99	1,22	.009	0.31				
Contour Tones: Learning	17.38	1,22	.001	0.44	Control	−1.12	0.13	[−1.39, −0.86]
					Target	−0.95	0.13	[−1.23, −0.67]
Level Tones: Learning	0.00	1,22	.100	0.00	Control	−1.03	0.13	[−1.29, −0.77]
					Target	−1.03	0.13	[−1.30, −0.76]
Tone Type × Learning × TTG	**7.56**	**1,22**	**.023**	**0.26**				
H/F: Tone Type × Learning	**12.58**	**1,11**	**.009**	**0.53**				
H/F, Contour Tones: Learning	**15.44**	**1,11**	**.005**	**0.58**	**Control**	−**1.03**	0.16	[−1.38, −0.69]
					**Target**	−**0.78**	0.19	[−1.21, −0.36]
Learning	6.83	1,22	.031	0.24	Control	−1.08	0.13	[−1.34, −0.72]
					Target	−0.99	0.13	[−1.26, −0.81]
Block	3.20	5,110	.041	0.13				
*Pairwise comparisons*								
*Block 1 vs Block 4: p = .016*					*Block 1*	−*1.17*	*0.14*	*[*−*1.46,* −*0.87]*
					*Block 4*	−*0.92*	*0.12*	*[*−*1.18,* −*0.67]*
Learning × Block	4.33	5,110	.007	0.16				
Control: Block	5.45	5,110	.002	0.20				
*Pairwise comparisons*								
*Block 1 vs Block 3: p = .024*					*Block 1*	−*1.31*	*0.16*	*[*−*1.46,* −*0.87]*
*Block 1 vs Block 4: p = .013*					*Block 3*	−*1.03*	*0.13*	*[*−*1.31,* −*0.75]*
*Block 1 vs Block 6: p = .022*					*Block 4*	−*0.97*	*0.11*	*[*−*1.18,* −*0.67]*
*Block 5 vs Block 6: p = .016*					*Block 5*	−*1.12*	*0.15*	*[*−*1.34,* −*0.77]*
					*Block 6*	−*0.90*	*0.14*	*[*−*1.23,* −*0.67]*

*Notes*: Significant interactions that had no significant follow‐up effects are excluded here but can be found in the Supporting Information S1. Descriptive statistics for each significant main effect and multiple comparison to the right. Important effects and interactions marked in bold. Pairwise comparisons for significant multi‐level main effects shown in italics. Greenhouse‐Geisser and FDR corrections applied. (TTG = Target Tone Group, H/F = high/fall learners).

#### 3.2.2 400–600 ms

**FIGURE 8:**
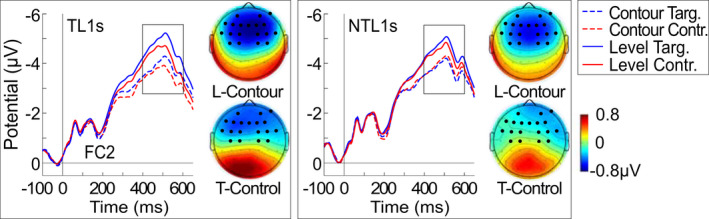
ERPs and subtraction topographies for the anterior negativity at 400–600 ms. ERPs at frontocentral electrode FC2 and topographies for the Tone Type effect (level‐contour; top) and the Learning effect (target‐control; bottom) for all trials in the tonal (TL1) and nontonal L1 (NTL1) group. Significant cluster electrodes marked in black. For better visibility, only part of the epoch and baseline are shown. The full epoch and more electrodes can be found in the Supporting Information S3. T = Target words; C = Control words; L = Level tones

For the second time window, permutation analysis produced two significant clusters for the comparison of words with contour tones and words with level tones for the first 20 minutes of the first session in all participants. There was a significant frontocentral cluster (AF3, AF4, AF8, F5, F3, F1, Fz, F2, F4, FC5, FC3, FC1, FC2, FC4, FC6, C3, C1, C2, C4), *p* < .001, *d* = 0.38, as well as a significant posterior cluster (FT7, FT8, TP7, CP6, TP8, P7, P5, P8, PO7, POz, PO8, Oz), *p <* .001, *d* = 0.33. See Figure 8.

A mixed ANOVA of the mean ERP amplitudes of the frontocentral cluster for all participants in 20‐minute blocks yielded a number of main effects and interactions for Tone Type, Learning, and temporal factors Block and Day, see Table 3 for details. With regards to Tone Type, level tones were more negative than contour tones. This difference was stronger in the tonal L1 group than in the nontonal L1 group. For Learning, we found that target words elicited larger negativities than control words (see Figure 8). The difference was again greater in the tonal L1 group and was also stronger on day 2 than on day 1. Finally, a general decrease of the negativity was observed over time.

**TABLE 3:** All significant results of the mixed Analysis of Variance analysis (ANOVA) for ERPs of the frontal cluster in the late time window

Effects and interactions	*F*	DF	*p*	$\eta_{p}^{2}$		Means in μV	Std error	95% Confidence interval
Tone Type	**191.83**	**1,44**	**<.001**	**0.81**	**Level**	−**3.26**	0.19	[−3.64, −2.88]
					**Contour**	−**2.80**	0.18	[−3.17, −2.47]
Learning	**36.73**	**1,44**	**<.001**	**0.46**	**Control**	−**2.89**	0.18	[−3.25, −2.52]
					**Target**	−**3.17**	0.19	[−3.56, −2.78]
Learning × Day	7.87	1,44	.015	0.15				
Day 1: Learning	20.55	1,44	<.001	0.32	Control	−3.16	0.20	[−3.56, −2.76]
					Target	−3.38	0.20	[−3.78, −2.98]
Day 2: Learning	38.55	1,44	<.001	0.47	Control	−2.61	0.18	[−2.97, −2.25]
					Target	−2.97	0.20	[−3.37, −2.56]
Tone Type × Learning × D × TTG	5.94	1,44	.038	0.12				
L/R: Tone Type × Learning × D	12.65	1,22	.004	0.37				
L/R, Level: Learning × D	14.53	1,22	.002	0.40				
L/R, Level, D2: Learning	23.97	1,22	<.001	0.53	Control	−2.69	0.25	[−3.20, −2.17]
					Target	−3.22	0.30	[−3.84, −2.61]
Day	**23.73**	**1,44**	**<.001**	**0.35**	**Day 1**	−**3.27**	0.20	[−3.67, −2.88]
					**Day 2**	−**2.79**	0.19	[−3.17, −2.41]

*Notes*: Significant interactions that had no significant follow‐up effects are excluded here but can be found in the Supporting Information S1. Descriptive statistics for each significant main effect and multiple comparison to the right. Important effects and interactions marked in bold. Pairwise comparisons for significant multi‐level main effects shown in italics. Greenhouse‐Geisser and FDR corrections applied. (TTG = Target Tone Group, H/F = high/fall learners, L/R = low/fall learners, D = Day).

For the posterior cluster, the effects were virtually indistinguishable from those of the frontocentral cluster but reversed in polarity: all of the main effects were near‐identical to those above, as were the crucial interaction clusters. Only two unique interactions emerged in the posterior cluster. We, therefore, chose to treat the positivity as a dipole effect and, for the sake of brevity, present the observed significant effects and interactions for the posterior positive cluster as Supporting Information S1 instead of in the main text.

#### 3.2.3 Correlation results

For the early effect, no significant correlations were found between changes in behavioral and neurophysiological data (*p* > .8). Further analysis revealed no significant correlations for the tonal learner’s H/F subgroup for this effect either (*p* > .2).

A significant correlation was observed between Amplitude Change for Anterior Negativity and Response Accuracy Change, *r* = −.353, *p* = .040, such that the larger the improvement in accuracy on day 1 was, the smaller the difference amplitude for the anterior negativity became. There was no significant correlation with Response Time Change (*p* > .7).

## 4 DISCUSSION

### 4.1 Word recognition component: Transfer effects

There was a clear effect of native language experience and familiarity in the pre‐attentive lexicality gating component at ~50 ms. The facilitation effect at this latency was only found at the highest degree of L1–L2 similarity. Our tonal learners did not show indications of facilitated word acquisition for all tonal target words or all target words with contour tones but instead only for target words with a falling tone. This became apparent in a reduced negativity which we assume reflects a successful, rapid word trace formation for target words with falling tones such that they became processed real‐word‐like already within the first 20 minutes of acquisition. A trend toward a similar amplitude decrease has previously been seen for real words in Mandarin speakers (Yue et al., 2014). After four minutes of word and legal pseudoword repetition, neural activity to real words appeared to become reduced. Note, however, that Yue et al. did not use an analysis that could cancel out frequency effects caused by the comparison of frequent standard and infrequent deviant stimuli (cf. Shtyrov & Lenzen, 2017) and, therefore, did not detect the amplitude change for real word deviants in their statistical analysis. Also, their time window was longer than ours. We mention the trend here, because Yue et al. (2014) is the only study to look at this early component in the context of tonal words. Notably, for words without tones, the same effect was previously reported in Kimppa et al. (2015). In accordance with these previous studies, we interpret the decreased effect size for narrowly L1‐facilitated target words as evidence that the words were acquired and processed like real words exceptionally quickly. Pseudowords, even those with the same pitch pattern, and nonfacilitated target words, on the other hand, could not be acquired equally rapidly and therefore evoked an increased negativity. This negativity likely signals an ongoing, incomplete memory trace formation process for untaught and nonfacilitated words.

Consistent with the idea of an ongoing word trace formation process, the negativity for both target words and control words decreased slightly over the course of the learning sessions (target words: *M*_B1_ = −1.02 μV ± SD = 0.8 μV, *M*_B6_ = −0.99 ± 0.8 μV; control words: *M*_B1_ = −1.31 ± 1.0 μV, *M_B6_ = −0.90 ± 0.8* μV). This decrease was significant only for the control words where the amplitude was highest initially. Interestingly, the decrease in amplitude proceeded in a step‐wise pattern for both word types such that the amplitude increased again after the breaks between blocks 2 and 3 and blocks 4 and 5 (cf. e.g., target words: *M*_B1_ = −1.02 μV ± SD = 0.8 μV, *M*_B2_ = −0.99 ± 0.9 μV, *M*_B3_ = −1.12±0.9 μV, *M*_B4_ = −0.88 ± 0.9 μV, *M*_B5_ = − 0.94± 0.8 μV, *M*_B6_ = −0.99 ± 0.8 μV). Together with the fact that we found no effect of learning session for the response amplitude at this latency, this suggests that word traces were only formed temporarily for words with nonnative phonology (i.e., tones). Thus, only words with a native phonology (and a familiar function) had a consistently reduced amplitude (*M* = 0.78 μV), which suggests that the rapid word trace formation process is dependent on the native neural phonology network and that word traces for L1‐like novel words were formed almost instantly and permanently.

The fact that we found a lasting facilitation effect only for target words with falling tones in the tonal learners is in accordance with previous studies which emphasized the importance of L1 tone shape (and function) in L2 tone perception (Burnham et al., 2015; Huang & Johnson, 2010). The effect further highlights the importance of falling tones and their association with inflections in the learners’ native language, Swedish. While it has previously been stipulated that learners of a contour tone language have facilitated perceptual access to contour tones overall (Gandour, 1983), this concept of general facilitation is not supported by the current data, at least for Swedish as L1. Instead, the present results indicate that L1 experience shaped L2 tone acquisition very narrowly, at least with respect to pre‐attentive lexical gating and memory trace formation. Thus, Swedish listeners only pre‐attentively responded to and differentiated L2 tones with the same pitch shape as tones of phonological importance at the word‐level in their native language, i.e., falls (Bruce, 1977, 1987, 2005). It is unclear whether the strict reliance on formal and functional similarity is universal or only holds for languages like Swedish, where only one type of tone movement has word‐level relevance. It is possible still that there is a generally heightened sensitivity for various tone movements in languages where different pitch shapes are phonologically contrastive and that Swedish simply does not compare to East‐Asian tone languages in this respect; this could be investigated in future cross‐linguistic studies. Furthermore, the L1 facilitation of L2 tone processing at the pre‐attentive level also required a high level of similarity in the tones’ higher‐level function. Processing of words with falling pitch was only facilitated when the tones had an inflectional function and not when they were presented in pseudowords. Similarly, the nontonal learners showed no facilitation although pitch falls are a common intonational pattern on monosyllables in their L1 (cf., Gibbon, 1998; Isačenko & Schädlich, 1970). It is likely that even learners with a lexical L1 tone would not have been sensitive to the tones’ inflectional function nor shown any facilitation effects, even if pitch shape had been similar.

Alternative to the lexical gating interpretation, the early effect could also be produced by more low‐level sensory processes. Thus, it could indicate a general attenuation of the neural response to the incoming auditory stimuli, much as is seen in the later time window. In the initial processing stages, such an attenuation could occur faster for stimuli that are more familiar to the listener—here, based on L1–L2 transfer. However, this suggestion lacks explanatory power, as it cannot easily account for the fact that only target words with the familiar pitch pattern but not control words with the same pitch pattern undergo attenuation. If this was a purely sensory response, it should not be affected by lexicality status.

### 4.2 Anterior negativity, response times, and accuracy: No transfer effects

While we found clear influences of language experience on the pre‐attentive processing of foreign tone, no such influences were visible at later, higher‐level processing stages or in behavioral responses. Regardless of native language background or type of target tones, all four subgroups were equally accurate and quick at detecting tone mismatches. Accuracy levels for (rule‐based) tone mismatch detection were relatively low (<70%) compared with (rule‐based) vowel mismatch detection and (lexical) consonant mismatch detection accuracies (>80%). That is, more than eight out of ten mismatches were noticed when the mismatch was based on vowels and consonants but less than seven out of ten mismatches when the mismatch was tone‐related. While only tone mismatch detection accuracy was part of the present analysis, we believe that the other two measures are important for indicating that participants could become very proficient at even overtly detecting rule‐based mismatches and gain fairly high accuracy levels overall. This attests to the general difficulty of L2 tone acquisition, likely due to underlying problems with the perception or classification of tones. There was a vast spread in response accuracy for tone mismatches, as apparent in Figure 5. The participants from the tonal H/F group had the overall highest accuracy (*M*_TL1_H/F_ = 77%; *SD* = 18 vs *M*_others_ < 62%; *SD* = >27) and the largest accuracy increase between days (*M*_TL1_H/F_ = 12%; *SD* = 10 vs *M*_others_ < 10%; *SD* = >13). Virtually none of the tonal participants’ accuracy decreased between days while a drop in accuracy was relatively common for the nontonal participants. One tonal and one nontonal participant had an accuracy of below 10 percent on day 2. Their responses indicated that they had deemed the tonal differences nonmeaningful and were unable to classify tone errors as such. This was confirmed during their debriefing after the experiment: Like all other participants, they had realized that the words could be separated into lexical and grammatical components but unlike the rest of the participants, they had only fully categorized the rule for the vowel contrast (e.g., a is singular, ɛ is plural), not the tone contrast (e.g., high is masculine, fall is feminine). While a certain amount of group‐based variation in response accuracy and change over time is apparent from Figure 5, there was also considerable intra‐group variability and response accuracy did not vary significantly as a factor of group.

While participants showed varying degrees of difficulty with offline tone error detection, they were able to use the tones online to differentiate between target and control words, that is, between real and pseudowords, visible in an increased anterior negativity (AN) to target words. This was presumably based on the words’ prominent grammatical content present in the tone as well as in the vowel change. The increased anterior negativity for the meaningful, double‐inflected target words compared with pseudowords is likely indicative of a larger processing cost during the rule‐based processing of the grammatical morphemes, similar to what was found in Krott and Lebib (2013) in the comparison of regular and irregular verbs. This is consistent with the traditional anterior negativity effect where grammar errors (typically related to agreement) are argued to increase the cost of rule‐based decomposition in real words (e.g., Krott et al., 2006; Krott & Lebib, 2013; Rodriguez‐Fornells et al., 2001; Schremm et al., 2019). In line with this argumentation, there was a decrease in amplitude difference between targets and controls over time, correlated with increased accuracy across participants. That is, for those learners whose accuracy increased most between the beginning and end of the first day of learning, the difference between targets and controls in the anterior negativity decreased most. This illustrates nicely that reduced amplitude for these stimuli was likely related to a reduced processing cost due to learning: The novel inflected words became easier to decompose over time as the rules became more entrenched, resulting in a reduction of the neural activity necessary for successful grammar processing. This is paralleled by a decrease in amplitudes on the second day of learning compared with the first day. Purely semantic associative training has previously produced a comparable finding: a decrease in the N400 due to repetition (Bermúdez‐Margaretto et al., 2018). The use of inflected novel words in the present study elicited a grammar‐related AN, but the concept of a reduced processing cost for initially novel words due to learning and entrenchment still holds true. Interestingly, the present study found the reduced amplitude on day 2 to be significant only for learners with high/fall target words, possibly due to a general trend for overnight consolidation effects, reinforced through substantial transfer‐facilitated consolidation in the high/fall group of the tonal L1 participants.

Besides being influenced by target and control word status, the amplitude of the anterior negativity was also impacted by tone type: the negativity was reduced for words with contour tones compared with words with level tones. This was the case in both learner groups irrespective of whether words were targets or controls. We suggest that contour tones, at least in the context of the present study, are more perceptually prominent than level tones, regardless of whether they are assigned meaning or not. We base this on the fact that, in the learning paradigm in our study, all target words had the same pitch onset and could be differentiated based on whether the pitch stayed at onset level (level tone) or started moving (contour tone). The same was true for control words. Thus, movement onset was a strong cue to word dissociation, which presumably made contour tones more perceptually prominent or salient than level tones. We thus interpret the observed decrease in anterior negativity as related to the contour tones’ high prominence level. This factor, maybe study‐specific, maybe general, likely reduced the overall processing load of contour tones compared with level tones, manifesting in a decrease in ERP amplitudes for the AN. Similar facilitation effects for acoustic properties of tones, outside of the context of grammar, have previously been reported in the comparison of high and low pitch (piano tones, Gosselke Berthelsen et al., 2018) and of tones with steep (high‐low) and moderate (mid‐low) falls (Swedish tones, Gosselke Berthelsen et al., 2018; Kochančikaitė et al., 2022). The high tones or high onsets in these studies resulted in reduced negativities, suggesting a relatively stronger salience of high pitch and/or steeper movement and thus less effortful processing. Hence, at least for the listener groups in the previous studies as well as in the present one (i.e., Swedes and Germans), certain general characteristics of tones appear to shape the tones’ perceptual prominence and, as a result, their processability. Interestingly, although perhaps unsurprisingly, the generally reduced processing cost is visible in the component that is most strongly involved with the processing of the given stimuli: a grammar‐related anterior negativity. The same reduced anterior negativity for perceptually prominent tones has been observed for the processing of native natural language with a focus on grammar associations (Gosselke Berthelsen et al., 2018). However, in grammar‐devoid contexts or for L2 learners that do not yet have a good grasp of L2 grammar, the reduction is visible more centrally (Gosselke Berthelsen et al., 2018). In strongly lexical‐semantic contexts, we would anticipate a general acoustically based difference in processability to be more posteriorly distributed.

While previous studies on more experienced L2 tone learners in natural acquisition contexts have found no consistent error‐related changes in the AN or N400 (Gosselke Berthelsen et al., 2018; Pelzl et al., 2019, 2021), the present study found an AN response that was considerably larger for inflected novel words than for uninflected pseudowords. Thus, the amplitude of the AN was modulated crucially by the existence of grammatical content, suggesting that learners used rule‐based, decompositional processes to assess the inflected L2 words, much like native speakers are thought to. The AN was further affected by the learning process and general entrenchment of the word forms, such that its amplitude decreased with familiarization over time and the specific increase for learned words was reduced upon successful learning. Finally, pitch prominence also affected the AN such that its amplitude was reduced for highly salient pitch patterns. All factors influencing the anterior negativity likely did so as they differentially affected the processing cost necessary to process the novel words. Thus, the AN component increased when words contained grammatical information and required decomposition, but the neural activity was reduced as processing became less resource‐heavy with successful learning (while error‐detection accuracy increased), with familiarization over time, and when words were easy to distinguish due to salient pitch features. While the quick emergence of L1‐like processing in L2 learners was certainly surprising considering previous tone learning literature, the lack of N400 or AN effects in previous studies might be explained by assuming that errors in the L2 initially do not significantly increase the already high, general cost of L2 processing (cf. Hahne & Friederici, 2001).

## 5 CONCLUSION

The current study investigated how native language experience shapes L2 tone processing and acquisition. We found a narrow effect of L1–L2 similarity in an early word recognition component at ~50 ms, such that only tones that were identical to tonal learners’ native tones in function and pitch shape were acquired ultra‐rapidly, i.e., within 20 minutes, and consequentially stood out against all other types of tones. For nontonal learners, there was no difference between tone types in the early, pre‐attentive ERPs, supporting the assumption that differential pre‐attentive processing and memory trace formation cannot occur without functional L1–L2 transfer. Later processing, which is modulated by attention, on the other hand, was not differentially affected by the learners’ language experience. All learners elicited an anterior negativity that was larger for inflected novel words than for meaningless pseudowords, indicative of rule‐based processing of the inflected words. This was facilitated through entrenchment and pitch prominence resulting in an amplitude reduction, suggesting that the overall processing cost decreased with learning and for the more salient contour tones. Similar to attention‐modulated processing, the learners’ behavioral responses to tone mismatches were not significantly affected by L1 experience. Both groups and all four subgroups identified tone mismatches equally rapidly and equally well. Thus, the present results suggest that pre‐attentive processing during L2 tone acquisition can be facilitated by language experience. At the same time, they relativize the importance of transfer effects, suggesting that only highly similar phonetic features in L1 and L2 lead to the facilitation of pre‐attentive processing. They further reveal that a lack of L1–L2 similarity can be overcome during later, higher‐level stages of speech processing which is likely an important reason why transfer effects do not always appear in studies on L2 acquisition and L2 processing. However, it is possible that the impact of pre‐attentive processing is stronger in natural second‐language acquisition and that learners, depending on testing conditions, can experience learning advantages for facilitated tones.

## CONFLICT OF INTEREST

The authors declare that there is no conflict of interest.

## AUTHOR CONTRIBUTIONS

**Sabine Gosselke Berthelsen:** Conceptualization; formal analysis; investigation; methodology; visualization; writing – original draft; writing – review and editing. **Merle Horne:** Conceptualization; supervision; writing – review and editing. **Yury Shtyrov:** Conceptualization; funding acquisition; methodology; supervision; writing – review and editing. **Mikael Roll:** Conceptualization; funding acquisition; methodology; supervision; writing – review and editing.

## Supporting information


**Supplementary Information S1** Tables with all results from the three mixed Analysis of Variance (ANOVA) analyses

**Supplementary Information S2** (a) Six electrode plots illustrating the early effect in the non‐tonal learners’ high/fall group. (b) Six electrode plots illustrating the early effect in the non‐tonal learners’ low/rise group

**Supplementary Information S3** Six electrode plots illustrating the anterior negativity. Divided by learner group

## Footnotes

^1^
The term “induce” is frequently used in the literature on Scandinavian word accents and, essentially, refers to a process of conditioning or assignment. Accent‐2‐inducing suffixes in Swedish carry a pre‐accenting lexical tone (accent 2). When they combine with a word stem, they assign their tone to the (preceding) syllable, if that syllable is stressed (Riad, 2012).

^2^
The occurrence of the N400 for grammatical tone here is likely due to the learner’s proficiency—beginner learners respond to L2 grammar errors with an N400 rather than grammatical ERP responses—coupled with the pictorial presentation of the mismatches.

^3^
In the context of the present study, we studied canonical words with tonal inflections. As the words contained both lexical and grammatical information and no violations were used to single in on either type of information, we could not predict with certainty whether participants would produce an N400 or a (L)AN. We could not know whether lexicosemantic or grammatical processes would be most strongly visible in the electrophysiological response and, therefore, avoid making strong predictions here.

## References

[psyp14042-bib-0001] Andersson, A. , Sayehli, S. , & Gullberg, M. (2019). Language background affects online word order processing in a second language but not offline. Bilingualism: Language and Cognition, 22(4), 802–825. 10.1017/S1366728918000573

[psyp14042-bib-0002] Banti, G. (1989). Two cushitic systems: Somali and Oromo nouns. In H. van der Hülst & N. Smith (Eds.), Autosegmental studies on pitch accent (pp. 11–50). Foris Publications.

[psyp14042-bib-0003] Benjamini, Y. , & Hochberg, Y. (1995). Controlling the false discovery rate: A practical and powerful approach to multiple testing. Journal of the Royal Statistical Society. Series B (Methodological), 57(1), 289–300. 10.2307/2346101

[psyp14042-bib-0004] Bermúdez‐Margaretto, B. , Beltrán, D. , Cuetos, F. , & Domínguez, A. (2018). Brain signatures of new (pseudo‐) words: Visual repetition in associative and non‐associative contexts. Frontiers of Human Neuroscience, 12, 354. 10.3389/fnhum.2018.00354/full PMC6131611 30233345

[psyp14042-bib-0005] Blomberg, F. , Roll, M. , Frid, J. , Lindgren, M. , & Horne, M. (2020). The role of affective meaning, semantic associates, and orthographic neighbours in modulating the N400 in single words. The Mental Lexicon, 15(2), 159–186. 10.1075/ml.19021.blo

[psyp14042-bib-0006] Boersma, P. (2001). Praat, a system for doing phonetics by computer. Glot International, 5(9/10), 341–345.

[psyp14042-bib-0007] Brown‐Schmidt, S. , & Canseco‐Gonzalez, E. (2004). Who do you love, your mother or your horse? An event‐related brain potential analysis of tone processing in Mandarin Chinese. Journal of Psycholinguistic Research, 33(2), 103–135. 10.1023/B:JOPR.0000017223.98667.10 15098511

[psyp14042-bib-0008] Bruce, G. (1977). Swedish word accents in sentence perspective. Gleerup.

[psyp14042-bib-0009] Bruce, G. (1983). Accentuation and timing in Swedish. Folia Linguistica, 17(1‐4), 221–238. 10.1515/flin.1983.17.1-4.221

[psyp14042-bib-0010] Bruce, G. (1987). How floating is focal accent? In K. Gregersen & H. Basbøll (Eds.), Nordic prosody IV: Papers from a symposium (pp. 41–49). Odense University Press.

[psyp14042-bib-0011] Bruce, G. (2005). Intonational prominence in varieties of Swedish revisited. In S.‐A. Jun (Ed.), Prosodic typology: The phonology of intonation and phrasing. Oxford University Press. 10.1093/acprof:oso/9780199249633.003.0015

[psyp14042-bib-0012] Burnham, D. , Kasisopa, B. , Reid, A. , Luksaneeyanawin, S. , Lacerda, F. , Attina, V. , Rattanasone, N. X. , Schwarz, I. C. , & Webster, D. (2015). Universality and language‐specific experience in the perception of lexical tone and pitch. Applied Psycholinguistics, 36, 1459–1491. 10.1017/S0142716414000496

[psyp14042-bib-0013] Chan, R. K. , & Leung, J. H. (2020). Why are lexical tones difficult to learn? Insights from the incidental learning of tone‐segment connections. Studies in Second Language Acquisition, 42, 33–59. 10.1017/S0272263119000482

[psyp14042-bib-0014] Chandrasekaran, B. , Krishnan, A. , & Gandour, J. T. (2007). Mismatch negativity to pitch contours is influenced by language experience. Brain Research, 1128, 148–156. 10.1016/j.brainres.2006.10.064 17125749 PMC4372203

[psyp14042-bib-0015] Chang, C. B. , & Bowles, A. (2015). Context effects on second‐language learning of tonal contrasts. The Journal of the Acoustical Society of America, 136(6), 3703–3716. 10.1121/1.4937612 26723326

[psyp14042-bib-0016] Chen, J. , Best, C. T. , & Antoniou, M. (2020). Native phonological and phonetic influences in perceptual assimilation of monosyllabic Thai lexical tones by Mandarin and Vietnamese listeners. Journal of Phonetics, 83, 101013. 10.1016/j.wocn.2020.101013

[psyp14042-bib-0017] Dittinger, E. , Barbaroux, M. , D'Imperio, M. , Jäncke, L. , Elmer, S. , & Besson, M. (2016). Professional music training and novel word learning: From faster semantic encoding to longer‐lasting word representations. Journal of Cognitive Neuroscience, 28(10), 1584–1602. 10.1162/jocn_a_00997 27315272

[psyp14042-bib-0018] Eberhard, D. M. , Simons, G. F. , & Fennig, C. D. (Eds.). (2020). Ethnologue: Languages of the world (23rd ed.). SIL International http://www.ethonologue.com

[psyp14042-bib-0019] Elert, C.‐C. (1972). Tonality in Swedish: Rules and a list of minimal pairs. De Gruyter Mouton.

[psyp14042-bib-0020] Ellis, N. C. , & Sagarra, N. (2011). Learned attention in adult second language acquisition: A replication and generalization study and meta‐analysis. Studies in Second Language Acquisition, 33(4), 589–624. 10.1017/S0272263111000325

[psyp14042-bib-0021] Ettlinger, M. , Morgan‐Short, K. , Faretta‐Stutenberg, M. , & Wong, P. C. (2016). The relationship between artificial and second language learning. Cognitive Science, 40, 822–847. 10.1111/cogs.12257 26201508 PMC4723295

[psyp14042-bib-0022] Gandour, J. (1983). Tone perception in Far Eastern languages. Journal of Phonetics, 11, 149–175. 10.1016/S0095-4470(19)30813-7

[psyp14042-bib-0023] Gibbon, D. (1998). Intonation in German. In D. Hirst & A. Di Cristo (Eds.), Intonation systems: A survey of twenty languages (pp. 78–95). Cambridge University Press.

[psyp14042-bib-0024] Gosselke Berthelsen, S. , Horne, M. , Brännström, K. J. , Shtyrov, Y. , & Roll, M. (2018). Neural processing of morphosyntactic tonal cues in second‐language learners. Journal of Neurolinguistics, 45, 60–78. 10.1016/j.jneuroling.2017.09.001

[psyp14042-bib-0025] Gosselke Berthelsen, S. , Horne, M. , Shtyrov, Y. , & Roll, M. (2020). Different neural mechanisms for rapid acquisition of words with grammatical tone in learners from tonal and non‐tonal backgrounds: ERP evidence. Brain Research, 1729, 146614. 10.1016/j.brainres.2019.146614 31862273

[psyp14042-bib-0026] Gosselke Berthelsen, S. , Horne, M. , Shtyrov, Y. , & Roll, M. (2021). Phonological transfer effects in novice learners: A learner's brain detects grammar errors only if the language sounds familiar. Bilingualism: Language and Cognition, 24(4), 656–669. 10.1017/s1366728921000134

[psyp14042-bib-0027] Gottfried, T. L. , & Suiter, T. L. (1997). Effect of linguistic experience on the identification of Mandarin Chinese vowels and tones. Journal of Phonetics, 25(2), 207–231. 10.1006/jpho.1997.0042

[psyp14042-bib-0028] Hahne, A. , & Friederici, A. D. (2001). Processing a second language: Late learners' comprehension mechanisms as revealed by event‐related brain potentials. Bilingualism: Language and Cognition, 4(2), 123–141. 10.1017/S1366728901000232

[psyp14042-bib-0029] Hed, A. , Schremm, A. , Horne, M. , & Roll, M. (2019). Neural correlates of second language acquisition of tone‐grammar associations. The Mental Lexicon, 14(1), 98–123. 10.1075/ml.17018.hed

[psyp14042-bib-0030] Herrmann, B. , Maess, B. , & Friederici, A. D. (2011). Violation of syntax and prosody ‐ Disentangling their contributions to the early left anterior negativity. Neuroscience Letters, 490, 116–120. 10.1016/j.neulet.2010.12.039 21185355

[psyp14042-bib-0031] Herrmann, B. , Maess, B. , Hahne, A. , Schröger, E. , & Friederici, A. D. (2011). Syntactic and auditory spatial processing in the human temporal cortex: An MEG study. NeuroImage, 57, 624–633. 10.1016/j.neuroimage.2011.04.034 21554964

[psyp14042-bib-0032] Herrmann, B. , Maess, B. , Hasting, A. S. , & Friederici, A. D. (2009). Localization of the syntactic mismatch negativity in the temporal cortex: An MEG study. NeuroImage, 48, 590–600. 10.1016/j.neuroimage.2009.06.082 19595773

[psyp14042-bib-0033] Ho, A. , Boshra, R. , Schmidtke, D. , Oralova, G. , Moro, A. L. , Service, E. , & Connolly, J. F. (2019). Electrophysiological evidence for the integral nature of tone in Mandarin spoken word recognition. Neurophysiologia, 131, 325–332. 10.1016/j.neuropsychologia.2019.05.031 31185227

[psyp14042-bib-0034] Hollingshead, A. B. (1975). Four factor index of social status. (Unpublished manuscript). Yale University.

[psyp14042-bib-0035] Hornscheidt, A. (2003). Linguistic and public attitudes towards gender in Swedish. In M. Hellinger & H. Bußmann (Eds.), Gender across languages: The linguistic representation of women and men (Vol. 3, pp. 339–368). John Benjamins.

[psyp14042-bib-0036] Huang, T. , & Johnson, K. (2010). Language specificity in speech perception: Perception of Mandarin tones by native and nonnative listeners. Phonetica, 67, 243–267. 10.1159/000327392 21525779 PMC7077082

[psyp14042-bib-0037] Hyman, L. M. (2009). How (not) to do phonological typology: The case of pitch‐accent. Language Sciences, 31, 213–238. 10.1016/j.langsci.2008.12.007

[psyp14042-bib-0038] Hyman, L. M. (2016). Lexical vs. grammatical tone: Sorting out the differences. Proceedings of Tonal Aspects of Languages, 2016, 6–11. 10.21437/TAL.2016-2

[psyp14042-bib-0039] Isačenko, A. , & Schädlich, H.‐J. (1970). A model of standard German intonation. In J. Pheby & H. Eras (Eds.), Janua Linguarum. Series Practica (Vol. 113). De Gruyter Mouton.

[psyp14042-bib-0040] ISO . (2004). ISO 389‐8. Acoustics: Reference zero for the calibration of audiometric equipment. Part 8: Reference equivalent threshold sound pressure levels for pure tone and circumaural earphones. International Organization for Standardization 389‐8.

[psyp14042-bib-0041] Jung, T.‐P. , Makeig, S. , Humphries, C. , Lee, T.‐W. , McKeown, M. J. , Iragui, V. , & Sejnowski, T. J. (2000). Removing electroencephalographic artifacts by blind source separation. Psychophysiology, 37, 163–178. 10.1111/1469-8986.3720163 10731767

[psyp14042-bib-0042] Kimppa, L. , Kujala, T. , Leminen, A. , Vainio, M. , & Shtyrov, Y. (2015). Rapid and automatic speech‐specific learning mechanism in human neocortex. NeuroImage, 118, 282–291. 10.1016/j.neuroimage.2015.05.098 26074199

[psyp14042-bib-0043] Kimppa, L. , Shtyrov, Y. , Hut, S. C. , Hedlund, L. , Leminen, M. , & Leminen, A. (2019). Acquisition of L2 morphology by adult language learners. Cortex, 116, 74–90. 10.1016/j.cortex.2019.01.012 30832994

[psyp14042-bib-0044] Kochančikaitė, R. , Shtyrov, Y. , & Roll, M. (2022). Lexical and decompositional processing in parallel? Evidence from Swedish, a pitch‐accent language. (Manuscript in preparation).

[psyp14042-bib-0045] Koriat, A. , & Greenberg, S. N. (1991). Syntactic control of letter detection: Evidence from English and Hebrew Nonwords. Journal of Experimental Psychology: Learning, Memory, and Cognition, 17(6), 1035–1050. 10.1037/0278-7393.17.6.1035

[psyp14042-bib-0046] Koriat, A. , Greenberg, S. N. , & Goldshmid, Y. (1991). The missing‐letter effect in Hebrew: Word frequency or word function? Journal of Experimental Psychology: Learning, Memory, and Cognition, 17(1), 66–80. 10.1037/0278-7393.17.1.66

[psyp14042-bib-0047] Krott, A. , Baayen, H. R. , & Hagoort, P. (2006). The nature of anterior negativities caused by misapplication of morphological rules. Journal of Cognitive Neuroscience, 18(10), 1616–1630. 10.1162/jocn.2006.18.10.1616 17014367

[psyp14042-bib-0048] Krott, A. , & Lebib, R. (2013). Electrophysiological evidence for a neural substrate of morphological rule application in correct wordforms. Brain Research, 1496, 70–83. 10.1016/j.brainres.2012.12.012 23246923 PMC3573228

[psyp14042-bib-0049] Kutas, M. , & Federmeier, K. D. (2011). Thirty years and counting: Finding meaning in the N400 component of the event related brain potential (ERP). Annual Review of Psychology, 62, 621–647. 10.1146/annurev.psych.093008.131123 PMC4052444 20809790

[psyp14042-bib-0050] Kutas, M. , & Hillyard, S. A. (1980). Reading senseless sentences: Brain potentials reflect semantic incongruity. Science, 207(4427), 203–205. 10.1126/science.7350657 7350657

[psyp14042-bib-0051] Ling, W. , & Grüter, T. (2020). From sounds to words: The relation between phonological and lexical processing of tone in L2 Mandarin. Second Language Research, 38(2), 289–313. 10.1177/0267658320941546

[psyp14042-bib-0052] Liu, C. (2013). Just noticeable difference of tone pitch contour change for English‐ and Chinese‐native listeners. The Journal of the Acoustical Society of America, 134(4), 3011–3020. 10.1121/1.4820887 24116436

[psyp14042-bib-0053] Liu, J. , & Wiener, S. (2020). Homophones facilitate lexical development in a second language. System, 91, 102249. 10.1016/j.system.2020.102249

[psyp14042-bib-0054] MacGregor, L. J. , Difrancesco, S. , Pulvermüller, F. , Shtyrov, Y. , & Mohr, B. (2015). Ultra‐rapid access to words in chronic aphasia: The effects of intensive language action therapy (ILAT). Brain Topography, 28, 279–291. 10.1007/s10548-014-0398-y 25403745 PMC4330459

[psyp14042-bib-0055] MacGregor, L. J. , Pulvermüller, F. , van Casteren, M. , & Shtyrov, Y. (2012). Ultra‐rapid access to words in the brain. Nature Communications, 3(711), 1:7. 10.1038/ncomms1715 PMC3543931 22426232

[psyp14042-bib-0056] Maddieson, I. (2013). Tone. In M. S. Dryer & M. Haspelmath (Eds.), The world atlas of language structures online. Max Planck Institute for Evolutionary Anthropology.

[psyp14042-bib-0057] Malins, J. G. , & Joanisse, M. F. (2012). Setting the tone: An ERP investigation of the influences of phonological similarity on spoken word recognition in Mandarin Chinese. Neuropsychologia, 50, 2032–2043. 10.1016/j.neuropsychologia.2012.05.002 22595659

[psyp14042-bib-0058] Maris, E. , & Oostenveld, R. (2007). Nonparametric statistical testing of EEG‐ and MEG‐data. Journal of Neuroscience Methods, 164, 177–190. 10.1016/j.jneumeth.2007.03.024 17517438

[psyp14042-bib-0059] McCarthy, G. , & Nobre, A. C. (1993). Modulation of semantic processing by spatial selective attention. Electroencephalography and Clinical Neurophysiology, 88, 210–219. 10.1016/0168-5597(93)90005-A 7684969

[psyp14042-bib-0060] Näätänen, R. , & Alho, K. (1995). Mismatch negativity—A unique measure of sensory processing in audition. International Journal of Neuroscience, 80(1‐4), 317–337. 10.3109/00207459508986107 7775056

[psyp14042-bib-0061] Näätänen, R. , Gaillard, A. W. , & Mäntysalo, S. (1978). Early selective‐attention effect on evoked potential reinterpreted. Acta Psychologica, 42(4), 313–329. 10.1016/0001-6918(78)90006-9 685709

[psyp14042-bib-0062] Okita, T. , & Jibu, T. (1998). Selective attention and N400 attenuation with spoken word repetition. Psychophysiology, 35(3), 260–271. 10.1017/s0048577298961042 9564746

[psyp14042-bib-0063] Osterhout, L. , & Mobley, L. A. (1995). Event‐related brain potentials elicited by failure to agree. Journal of Memory and Language, 34(6), 739–773. 10.1006/jmla.1995.1033

[psyp14042-bib-0064] Partanen, E. , Leminen, A. , de Paoli, S. , Bundgaard, A. , Kingo, O. S. , Krøjgaard, P. , & Shtyrov, Y. (2017). Flexible, rapid and automatic neocortical word form acquisition mechanism in children as revealed by neuromagnetic brain response dynamics. NeuroImage, 155, 450–459. 10.1016/j.neuroimage.2017.03.066 28389383

[psyp14042-bib-0065] Pelzl, E. , Lau, E. F. , Guo, T. , & DeKeyser, R. (2019). Advanced second language learners' perception of lexical tone contrasts. Studies in Second Language Acquisition, 41, 59–86. 10.1017/S0272263117000444

[psyp14042-bib-0066] Pelzl, E. , Lau, E. F. , Guo, T. , & DeKeyser, R. (2021). Even in the best‐case scenario L2 learners have persistent difficulty perceiving and utilizing tones in Mandarin: Findings from behavioural and event‐related potentials experiments. Studies in Second Language Acquisition, 43(2), 268–296. 10.1017/S027226312000039X

[psyp14042-bib-0067] Qin, Z. , & Mok, P. P.‐K. (2011). Discrimination of Cantonese tones by Mandarin, English and French speakers. *Conference: The Psycholinguistic representation of tone conference (PLRT)*.

[psyp14042-bib-0068] Riad, T. (2012). Culminativity, stress and tone accent in Central Swedish. Lingua, 122, 1352–1379. 10.1016/j.lingua.2012.07.001

[psyp14042-bib-0069] Riad, T. (2014). The phonology of Swedish. Oxford University Press.

[psyp14042-bib-0070] Rischel, J. (1963). Morphemic tone and word tone in Eastern Norwegian. Phonetica, 10, 154–164. 10.1159/000258166

[psyp14042-bib-0071] Rodriguez‐Fornells, A. , Clahsen, H. , Lleó, C. , Zaake, W. , & Münte, T. F. (2001). Event‐related brain responses to morphological violations in Catalan. Cognitive Brain Research, 11(1), 47–58. 10.1016/S0926-6410(00)00063-X 11240111

[psyp14042-bib-0072] Roll, M. (2015). A neurolinguistic study of South Swedish word accents: Electrical brain potentials in nouns and verbs. Nordic Journal of Linguistics, 38(2), 149–162. 10.1017/S0332586515000189

[psyp14042-bib-0073] Roll, M. , Söderström, P. , & Horne, M. (2013). Word‐stem tones cue suffixes in the brain. Brain Research, 1520, 116–120. 10.1016/j.brainres.2013.05.013 23685193

[psyp14042-bib-0074] Schremm, A. , Novén, M. , Horne, M. , & Roll, M. (2019). Brain responses to morphologically complex verbs: An electrophysiological study of Swedish regular and irregular past tense forms. Journal of Neurolinguistics, 51, 76–83. 10.1016/j.jneuroling.2019.01.006

[psyp14042-bib-0075] Schremm, A. , Novén, M. , Horne, M. , Söderström, P. , van Westen, D. , & Roll, M. (2018). Cortical thickness of planum temporale and pars opercularis in native language tone processing. Brain and Language, 176, 42–47. 10.1016/j.bandl.2017.12.001 29223785

[psyp14042-bib-0076] Shen, G. , & Froud, K. (2019). Electrophysiological correlates of categorical perception of lexical tones by English learners of Mandarin Chinese: An ERP study. Bilingualism: Language and Cognition, 22(2), 253–265. 10.1017/S136672891800038X

[psyp14042-bib-0077] Shtyrov, Y. , & Lenzen, M. (2017). First‐pass neocortical processing of spoken language takes only 30 msec: Electrophysiological evidence. Cognitive Neuroscience, 8(1), 24–38. 10.1080/17588928.2016.1156663 26919206

[psyp14042-bib-0078] So, C. K. , & Best, C. T. (2014). Phonetic influences on English and French listeners' assimilation of Mandarin tones to native prosodic categories. Studies in Second Language Acquisition, 36, 195–221. 10.1017/S0272263114000047

[psyp14042-bib-0079] Söderström, P. , Horne, M. , & Roll, M. (2017). Stem tones pre‐activate suffixes in the brain. Journal of Psycholinguistic Research, 46(2), 371–380. 10.1007/s10936-016-9434-2 PMC5368231 27240896

[psyp14042-bib-0080] Unsworth, N. , Heitz, R. P. , Schrock, J. C. , & Engle, R. W. (2005). An automated version of the operation span task. Behavior Research Methods, 37(3), 498–505. 10.3758/BF03192720 16405146

[psyp14042-bib-0081] Wong, P. C. , & Perrachione, T. K. (2007). Learning pitch patterns in lexical identification by native English‐speaking adults. Applied Psycholinguistics, 28, 565–585. 10.1017/S0142716407070312

[psyp14042-bib-0082] Yip, M. J. (2002). Tone. Cambridge University Press.

[psyp14042-bib-0083] Yu, K. , Li, L. , Chen, Y. , Zhou, Y. , Wang, R. , Zhang, Y. , & Li, P. (2019). Effects of native language experience on Mandarin lexical tone processing in proficient second language learners. Psychophysiology, 56, e13448. 10.1111/psyp.13448 31355474

[psyp14042-bib-0084] Yu, Y. H. , Shafer, V. L. , & Sussman, E. S. (2017). Neurophysiological and behavioral responses of Mandarin lexical tone processing. Frontiers in Neuroscience, 11. 10.3389/fnins.2017.00095 PMC5338334 28321179

[psyp14042-bib-0085] Yue, J. , Bastiaanse, R. , & Alter, K. (2014). Cortical plasticity induced by rapid Hebbian learning of novel tonal word‐forms: Evidence from mismatch negativity. Brain & Language, 139, 10–22. 10.1016/j.bandl.2014.09.007 25463813

[psyp14042-bib-0086] Zhao, J. , Guo, J. , Zhou, F. , & Shu, H. (2011). Time course of Chinese monosyllabic spoken word recognition: Evidence from ERP analyses. Neuropsychologia, 49, 1761–1770. 10.1016/j.neuropsychologia.2011.02.054 21382389

