# Supplementary material for: Native language experience shapes pre‐attentive foreign tone processing and guides rapid memory trace build‐up: An ERP study
Source: Psychophysiology. 2022 Mar 16;59(8):e14042. doi: 10.1111/psyp.14042 (PMC9539634; doi:10.1111/psyp.14042)
Supplement: Supplementary file 1 — Supplementary Information S1 Tables with all results from the three mixed Analysis of Variance (ANOVA) analyses [file PSYP-59-e14042-s002.pdf]

Supplementary material 1

Tables with all results from the three mixed Analysis of Variance (ANOVA) analyses.

Important abbreviations

LG = Learner Group
TTG = Target Tone Group
D = Day
B = Block
TL1s = tonal L1 learner group
NTL1s = non-tonal L1 learner group
H/F = high/fall group
L/R = low/rise group

Table 1.1: *Mixed ANOVA for the early time windows. Tonal L1 learners only. Descriptive statistics added for each significant main effect and multiple comparison after significant main effects for the non-binary factor 'Block'. Significant results marked in bold and with grey background.*

Effects and interactions	F	DF	p	η_p^2		Means in µV	Std Error	95% Confidence Interval
Tone Type	0.04	1,22	1.000	0.00				
Tone Type * TTG	0.16	1,22	1.000	0.01				
**Learning**	**6.83**	**1,22**	**.031**	**0.24**	Control	-1.08	0.13	[-1.34, -0.72]
					Target	-0.99	0.13	[-1.26, -0.81]
Learning * TTG	0.00	1,22	1.000	0.00				
Day	0.13	1,22	1.000	0.01				
Day * TTG	3.37	1,22	.160	0.14				
**Block**	**3.20**	**5,110**	**.041**	**0.13**				
Pairwise comparisons:								
Block 1 vs Block 4: p = .016					*Block 1*	*-1.17*	*0.14*	*[-1.46, -0.87]*
					Block 4	*-0.92*	*0.12*	*[-1.18, -0.67]*
Block * TTG	0.98	5,110	.845	0.04				
**Tone Type * Learning**	**9.99**	**1,22**	**.009**	**0.31**				
Contour Tones: Learning	**17.38**	**1,22**	**.001**	**0.44**	Control	-1.12	0.13	[-1.39, -0.86]
					Target	-0.95	0.13	[-1.23, -0.67]
Level Tones: Learning	0.00	1,22	.100	0.00	Control	-1.03	0.13	[-1.29, -0.77]
					Target	-1.03	0.13	[-1.30, -0.76]
**Tone Type * Learning * TTG**	**7.56**	**1,22**	**.023**	**0.26**				
H/F: Tone Type * Learning	**12.58**	**1,22**	**.009**	**0.36**				
H/F, Contour Tones: Learning	**115.44**	**1,22**	**.005**	**0.84**	Control	-1.03	0.16	[-1.38, -0.69]
					Target	-0.78	0.19	[-1.21, -0.36]
H/F, Level Tones: Learning	1.15	1,22	.615	0.05	Control	-0.87	0.18	[-1.27, -0.48]
					Target	-0.95	0.19	[-1.37, -0.53]
L/R: Tone Type * Learning	0.14	1,22	1.000	0.00				
Tone Type * Day	0.77	1,22	.780	0.03				
Tone Type * Day * TTG	0.11	1,22	1.000	0.01				
Learning * Day	0.07	1,22	1.000	0.00				
Learning * Day * TTG	0.42	1,22	1.000	0.02				
Tone Type * Learning * Day	2.32	1,22	.285	0.10				
Tone Type * Learning * Day * TTG	0.36	1,22	1.000	0.02				
Tone Type * Block	1.08	5,110	.740	0.05				
Tone Type * Block * TTG	2.13	5,110	.174	0.09				

Effects and interactions	F	DF	p	η_p^2		Means in µV	Std Error	95% Confidence Interval
Learning * Block	**4.33**	**5,110**	**.007**	**0.16**				
Target: Block	1.71	5,110	.309	0.07				
Control: Block	**5.45**	**5,110**	**.002**	**0.20**				
Pairwise comparisons:								
Block 1 vs Block 3: p = .024					*Block 1*	*-1.17*	*0.14*	*[-1.46, -0.87]*
Block 1 vs Block 4: p = .013					*Block 3*	*-1.08*	*0.14*	*[-1.31, -0.75]*
Block 1 vs Block 6: p = .022					*Block 4*	*-0.92*	*0.12*	*[-1.18, -0.67]*
Block 5 vs Block 6: p = .015					*Block 5*	*-1.06*	*0.14*	*[-1.34, -0.77]*
Learning * Block * TTG	0.44	5,110	1.000	0.02				
Tone Type * Learning * Block	1.07	5,110	.748	0.05				
Tone Type * Learning * Block * TTG	1.13	5,110	.697	0.05				
Day * Block	0.31	5,110	1.000	0.01				
Day * Block * TTG	1.00	5,110	.820	0.04				
Tone Type * Day * Block	1.55	5,110	.396	0.07				
Tone Type * Day * Block * TTG	0.71	5,110	1.000	0.03				
Learning * Day * Block	0.97	5,110	.853	0.04				
Learning * Day * Block * TTG	1.58	5,110	.374	0.07				
Tone Type * Learning * D * B	0.98	5,110	.846	0.04				
Tone Type * Learning * D * B * TTG	1.45	5,110	.447	0.06				

Table 2.1: *All results of the mixed Analysis of Variance (ANOVA) analysis for ERPS of the frontal cluster in the late time window. Descriptive statistics added for each significant main effect and multiple comparison after significant main effects for the non-binary factor 'Block'. Significant results marked in bold and with grey background.*

Effects and interactions	F	DF	p	η_p^2		Means in µV	Std Error	95% Confidence Interval
Tone Type	**191.83**	**1,44**	**<.001**	**0.81**	Level	-3.26	0.19	[-3.64, -2.88]
					Contour	-2.80	0.18	[-3.17, -2.47]
Tone Type * LG	4.23	1,44	.092	0.09				
Tone Type * TTG	1.28	1,44	.528	0.03				
Tone Type * LG * TTG	0.63	1,44	.864	0.01				
Learning	**36.73**	**1,44**	**<.001**	**0.45**	Control	-2.89	0.18	[-3.25, -2.52]
					Target	-3.17	0.19	[-3.56, -2.78]
Learning * LG	4.37	1,44	.085	0.09				
Learning * TTG	0.32	1,44	1.000	0.01				
Learning * LG * TTG	2.22	1,44	.287	0.05				
Day	**23.73**	**1,44**	**<.001**	**0.35**	Day 1	-3.27	0.20	[-3.67, -2.88]
					Day 2	-2.79	0.19	[-3.17, -2.41]
Day * LG	0.19	1,44	1.000	0.00				
Day * TTG	5.23	1,44	.054	0.11				
Day * LG * TTG	0.44	1,44	1.000	0.01				
Block	**3.47**	**5,220**	**.025**	**0.07**				
no significant pairwise comparison								
Block * LG	1.77	5,220	.290	0.04				
Block * TTG	1.64	5,220	.346	0.04				
Block * LG * TTG	1.23	5,220	.601	0.03				
Tone Type * Learning	3.76	1,44	.118	0.08				
Tone Type * Learning * LG	0.04	1,44	1.000	0.00				
Tone Type * Learning * TTG	0.62	1,44	.874	0.01				
Tone Type * Learning * LG * TTG	0.22	1,44	1.000	0.00				
Tone Type * Day	0.01	1,44	1.000	0.00				
Tone Type * Day * LG	0.00	1,44	1.000	0.00				
Tone Type * Day * TTG	0.06	1,44	1.000	0.00				
Tone Type * Day * LG * TTG	0.87	1,44	.707	0.02				
Learning * Day	**7.87**	**1,44**	**.015**	**0.15**				
Day 1: Learning	**20.55**	**1,44**	**<.001**	**0.32**	Control	-3.16	0.20	[-3.56, -2.76]
					Target	-3.38	0.20	[-3.78, -2.98]
Day 2: Learning	**38.55**	**1,44**	**<.001**	**0.47**	Control	-2.61	0.18	[-2.97, -2.25]
					Target	-2.97	0.20	[-3.37, -2.56]
Learning * Day * LG	0.27	1,44	1.000	0.01				
Learning * Day * TTG	0.17	1,44	1.000	0.00				
Learning * Day * LG * TTG	0.91	1,44	.689	0.02				
Tone Type * Learning * Day	2.58	1,44	.231	0.06				
Tone Type * Learning * Day * LG	3.46	1,44	.139	0.07				
Tone Type * Learning * Day * TTG	**5.94**	**1,44**	**.038**	**0.12**				
L/R: Tone Type * Learning * Day	**12.65**	**1,22**	**.004**	**0.37**				
L/R, Contour: Learning * Day	0.00	1,22	1.000	0.00				
L/R, Level: Learning * Day	**14.53**	**1,22**	**.002**	**0.40**				
L/R, Level, D1: Learning	5.49	1,22	.057	0.20				
L/R, Level, D2: Learning	**23.97**	**1,22**	**<.001**	**0.52**	Control	-2.69	0.25	[-3.20, -2.17]
					Target	-3.22	0.30	[-3.84, -2.61]
H/F: Tone Type * Learning * Day	0.56	1,22	1.000	0.02				
Tone Type * Learning * D * LG * TTG	0.28	1,44	1.000	0.01				
Tone Type * Block	0.49	5,220	1.000	0.01				
Tone Type * Block * LG	**3.01**	**5,220**	**.029**	**0.06**				
no significant follow-up effects for this interaction								
Tone Type * Block * TTG	0.81	5,220	1.000	0.02				
Tone Type * Block * LG * TTG	**2.91**	**5,220**	**.035**	**0.06**				
no significant follow-up effects for this interaction								

Effects and interactions	F	DF	p	η_p^2	Means in µV	Std Error	95% Confidence Interval
Learning * Block	1.97	5,220	.205	0.04			
Learning * Block * LG	3.09	5,220	.051	0.07			
Learning * Block * TTG	1.03	5,220	.786	0.02			
Learning * Block * LG * TTG	1.31	5,220	.536	0.03			
Tone Type * Learning * B	0.70	5,220	1.000	0.02			
Tone Type * Learning * B * LG	1.06	5,220	.762	0.02			
Tone Type * Learning * B * LG	2.23	5,220	.122	0.05			
Tone Type * Learning * B * LG * TTG	0.74	5,220	1.000	0.02			
Day * Block	1.92	5,220	.256	0.04			
Day * Block * LG	0.80	5,220	.995	0.02			
Day * Block * TTG	2.26	5,220	.165	0.05			
Day * Block * LG * TTG	**3.30**	**5,220**	**.043**	**0.07**			
TL1: Day * Block * TTG	**4.19**	**5,110**	**.021**	**0.16**			
no significant follow-up effects for this interaction							
NTL1: Day * Block * TTG	1.17	5,110	.326	0.05			
Tone Type * Day * Block	1.47	5,220	.419	0.03			
Tone Type * Day * Block * LG	1.61	5,220	.344	0.04			
Tone Type * Day * Block * TTG	0.58	5,220	1.000	0.01			
Tone Type * Day * Block * LG * TTG	**3.09**	**5,220**	**.031**	**0.07**			
no significant follow-up effects for this interaction							
Learning * Day * Block	0.76	5,220	1.000	0.02			
Learning * Day * Block * LG	0.79	5,220	1.000	0.02			
Learning * Day * Block * TTG	0.40	5,220	1.000	0.01			
Learning * Day * Block * LG * TTG	1.52	5,220	.386	0.03			
Tone Type * Learning *D*B	0.28	5,220	1.000	0.01			
Tone Type * Learning *D*B* LG	1.12	5,220	.702	0.02			
Tone Type * Learning *D*B* TTG	0.95	5,220	.890	0.02			
Tone Type * Learning *D*B*LG*TTG	2.26	5,220	.117	0.05			

Table 2.2: *All results of the mixed Analysis of Variance (ANOVA) analysis for ERPS of the posterior cluster in the late time window. Descriptive statistics added for each significant main effect and multiple comparison after significant main effects for the non-binary factor 'Block'. Significant results marked in bold and with grey background.*

Effects and interactions	F	DF	p	η_p^2		Means in µV	Std Error	95% Confidence Interval
Tone Type	**183.35**	**1,44**	**<.001**	**0.81**	Level	2.21	0.15	[1.91, 2.50]
					Contour	1.86	0.14	[1.58, 2.15]
Tone Type * LG	4.43	1,44	.082	0.09				
Tone Type * TTG	2.15	1,44	.299	0.05				
Tone Type * LG * TTG	0.01	1,44	1.000	0.00				
Learning	**30.76**	**1,44**	**<.001**	**0.41**	Control	1.93	0.14	[1.84, 2.44]
					Target	2.14	0.15	[1.65, 2.22]
Learning * LG	3.13	1,44	.168	0.07				
Learning * TTG	0.26	1,44	1.000	0.01				
Learning * LG * TTG	1.87	1,44	.358	0.04				
Day	**24.10**	**1,44**	**<.001**	**0.35**	Day 1	2.22	0.16	[1.90, 2.53]
					Day 2	1.85	0.14	[1.57, 2.14]
Day * LG	0.19	1,44	1.000	0.00				
Day * TTG	**5.55**	**1,44**	**.046**	**0.11**				
L/R: Day	2.31	1,22	.285	0.10				
H/F: Day	**44.76**	**1,22**	**<.001**	**0.67**	Day 1	2.47	0.25	[1.95, 2.99]
					Day 2	1.93	0.21	[1.49, 2.37]
Day * LG * TTG	0.32	1,44	1.000	0.01				
Block	**3.16**	**5,220**	**.025**	**0.07**				
no significant pairwise comparison								
Block * LG	1.50	5,220	.426	0.03				
Block * TTG	1.91	5,220	.251	0.04				
Block * LG * TTG	0.84	5,220	.607	0.02				
Tone Type * Learning	2.98	1,44	.182	0.06				
Tone Type * Learning * LG	0.00	1,44	1.000	0.00				
Tone Type * Learning * TTG	0.15	1,44	1.000	0.00				
Tone Type * Learning * LG * TTG	0.18	1,44	1.000	0.00				
Tone Type * Day	0.11	1,44	1.000	0.00				
Tone Type * Day * LG	0.00	1,44	1.000	0.00				
Tone Type * Day * TTG	0.02	1,44	1.000	0.00				
Tone Type * Day * LG * TTG	1.43	1,44	.477	0.03				
Learning * Day	3.60	1,44	.129	0.08				
Learning * Day * LG	1.04	1,44	.625	0.02				
Learning * Day * TTG	0.22	1,44	1.000	0.00				
Learning * Day * LG * TTG	1.32	1,44	.513	0.03				
Tone Type * Learning * Day	0.69	1,44	.819	0.02				
Tone Type * Learning * Day * LG	4.84	1,44	.066	0.10				
Tone Type * Learning * Day * TTG	**6.37**	**1,44**	**.031**	**0.13**				
L/R: Tone Type * Learning * Day	**8.89**	**1,22**	**.014**	**0.29**				
L/R, D1: Tone Type * Learning	0.00	1,22	1.000	0.00				
L/R, D2: Tone Type * Learning	**8.80**	**1,22**	**.014**	**0.29**				
L/R, D2, Contour: Learning	**5.96**	**1,11**	**.046**	**0.35**	Control	1.54	0.20	[1.62, 2.44]
					Target	1.71	0.19	[1.81, 2.61]
L/R, D2, Level: Learning	**17.79**	**1,11**	**.001**	**0.62**	Control	1.75	0.17	[1.39, 2.11]
					Target	2.10	0.21	[1.67, 2.53]
H/F: Tone Type * Learning * Day	1.05	1,22	.317	0.05				
Tone Type * Learning * Day *LG*TTG	0.56	1,44	.914	0.01				
Tone Type * Block	1.01	5,220	.816	0.02				
Tone Type * Block * LG	1.74	5,220	.266	0.04				
Tone Type * Block * TTG	0.77	5,220	1.000	0.02				
Tone Type * Block * LG * TTG	2.18	5,220	.129	0.05				
Learning * Block	2.02	5,220	.184	0.04				
Learning * Block * LG	2.32	5,220	.115	0.05				
Learning * Block * TTG	0.70	5,220	1.000	0.02				
Learning * Block * LG * TTG	1.37	5,220	.490	0.03				

Effects and interactions	F	DF	p	η_p^2		Means in µV	Std Error	95% Confidence Interval
Tone Type * Learning * B	1.06	5,220	.768	0.02				
Tone Type * Learning * B * LG	1.26	5,220	.566	0.03				
Tone Type * Learning * B * TTG	1.97	5,220	.181	0.04				
Tone Type * Learning * B * LG * TTG	0.90	5,220	.947	0.02				
Day * Block	2.23	5,220	.173	0.05				
Day * Block * LG	0.75	5,220	1.000	0.02				
Day * Block * TTG	3.02	5,220	.062	0.06				
Day * Block * LG * TTG	3.14	5,220	.053	0.07				
Tone Type * Day * Block	1.52	5,220	.390	0.03				
Tone Type * Day * Block * LG	1.56	5,220	.366	0.03				
Tone Type * Day * Block * TTG	1.16	5,220	.662	0.03				
Tone Type * Day * Block * LG * TTG	**3.89**	5,220	**.007**	**0.08**				
L/R: Tone Type * Day * B * LG	1.34	5,110	.519	0.06				
H/F: Tone Type * Day * B * LG	**3.76**	**5,110**	**.013**	**0.15**				
H/F, D1: Tone Type * B * LG	**3.34**	**5,110**	**.030**	**0.13**				
H/F, D1, B1: Tone Type * LG	0.03	1,22	1.000	0.00				
H/F, D1, B2: Tone Type * LG	4.70	1,22	.082	0.18				
H/F, D1, B3: Tone Type * LG	3.09	1,22	.186	0.12				
H/F, D1, B4: Tone Type * LG	1.12	1,22	.601	0.05				
H/F, D1, B5: Tone Type * LG	2.13	1,22	.317	0.09				
H/F, D1, B6: Tone Type * LG	**7.86**	**1,22**	**.021**	**0.26**				
H/F, D1, B6, TL1: Tone Type	**34.52**	**1,11**	**<.001**	**0.76**	Level	2.73	0.38	[1.90, 3.57]
					Contour	2.13	0.38	[1.30, 2.95]
H/F, D1, B6, NTL1: Tone Type	0.84	1,11	.761	0.07				
H/F, D2: Tone Type * B * LG	2.60	5,110	.087	0.04				
Learning * Day * Block	1.04	5,220	.785	0.02				
Learning * Day * Block * LG	0.76	5,220	1.000	0.02				
Learning * Day * Block * TTG	0.56	5,220	1.000	0.01				
Learning * Day * Block * LG * TTG	1.62	5,220	.333	0.04				
Tone Type * Learning * D*B	0.12	5,220	1.000	0.00				
Tone Type * Learning * D*B*LG	1.12	5,220	.702	0.02				
Tone Type * Learning * D*B*TTG	1.30	5,220	.542	0.03				
Tone Type * Learning * D*B*LG*TTG	1.71	5,220	.295	0.04				

[1] FDR corrected.
