# Supplementary material for: Native language experience shapes pre‐attentive foreign tone processing and guides rapid memory trace build‐up: An ERP study
Source: Psychophysiology. 2022 Mar 16;59(8):e14042. doi: 10.1111/psyp.14042 (PMC9539634; doi:10.1111/psyp.14042)

Supplementary material 2a:

Six electrode plots illustrating the early effect in the non-tonal learners' high/fall group.

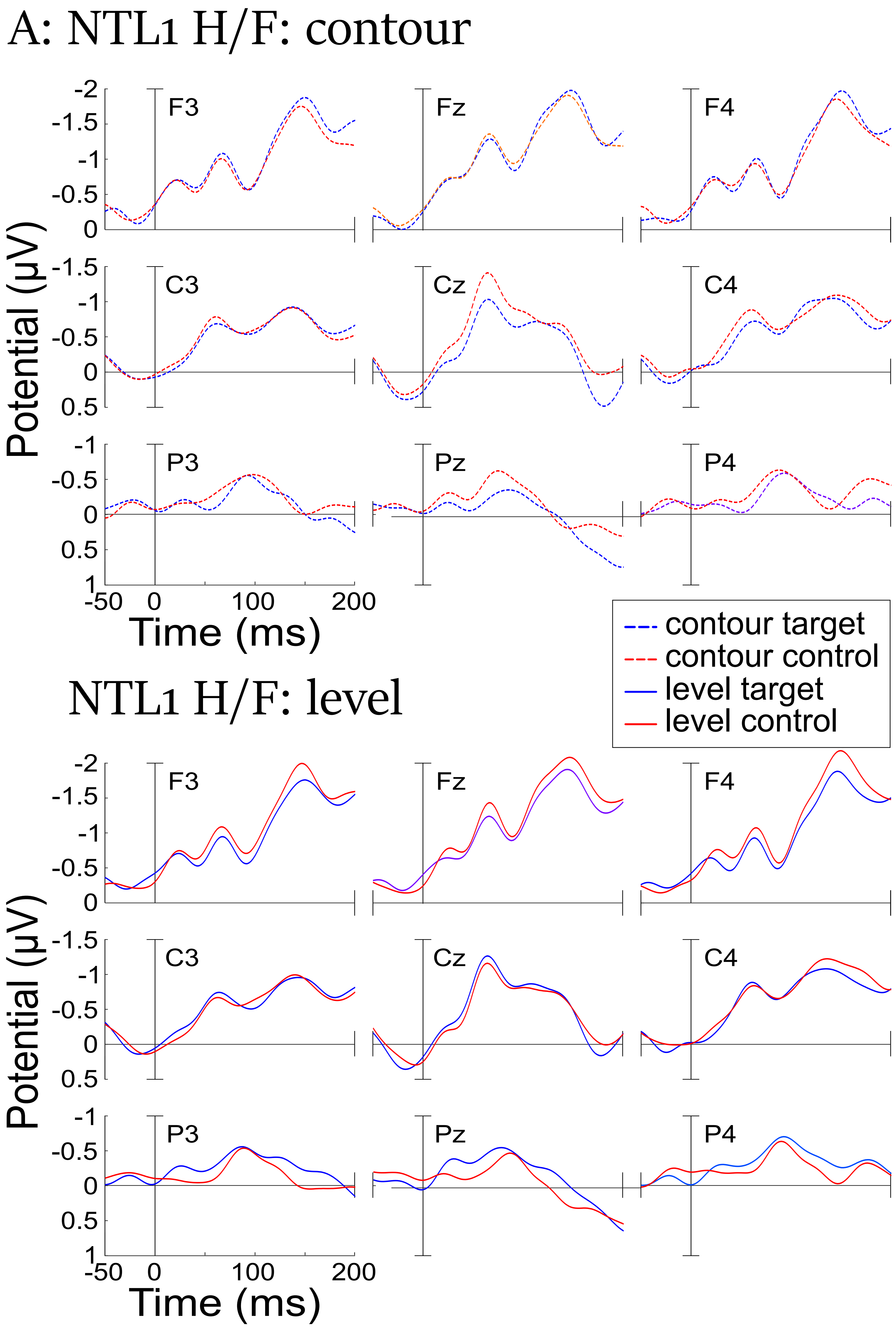

Supplement: Supplementary file 2 — Supplementary Information S2 (a) Six electrode plots illustrating the early effect in the non‐tonal learners’ high/fall group. (b) Six electrode plots illustrating the early effect in the non‐tonal learners’ low/rise group [file PSYP-59-e14042-s003.zip › psyp14042-sup-0002-SupplementarymaterialS2a.pdf]
