# Supplementary material for: Native language experience shapes pre‐attentive foreign tone processing and guides rapid memory trace build‐up: An ERP study
Source: Psychophysiology. 2022 Mar 16;59(8):e14042. doi: 10.1111/psyp.14042 (PMC9539634; doi:10.1111/psyp.14042)

Supplementary material 3:
Six electrode plots illustrating the anterior negativity. Divided by learner group.

A: tonal speakers

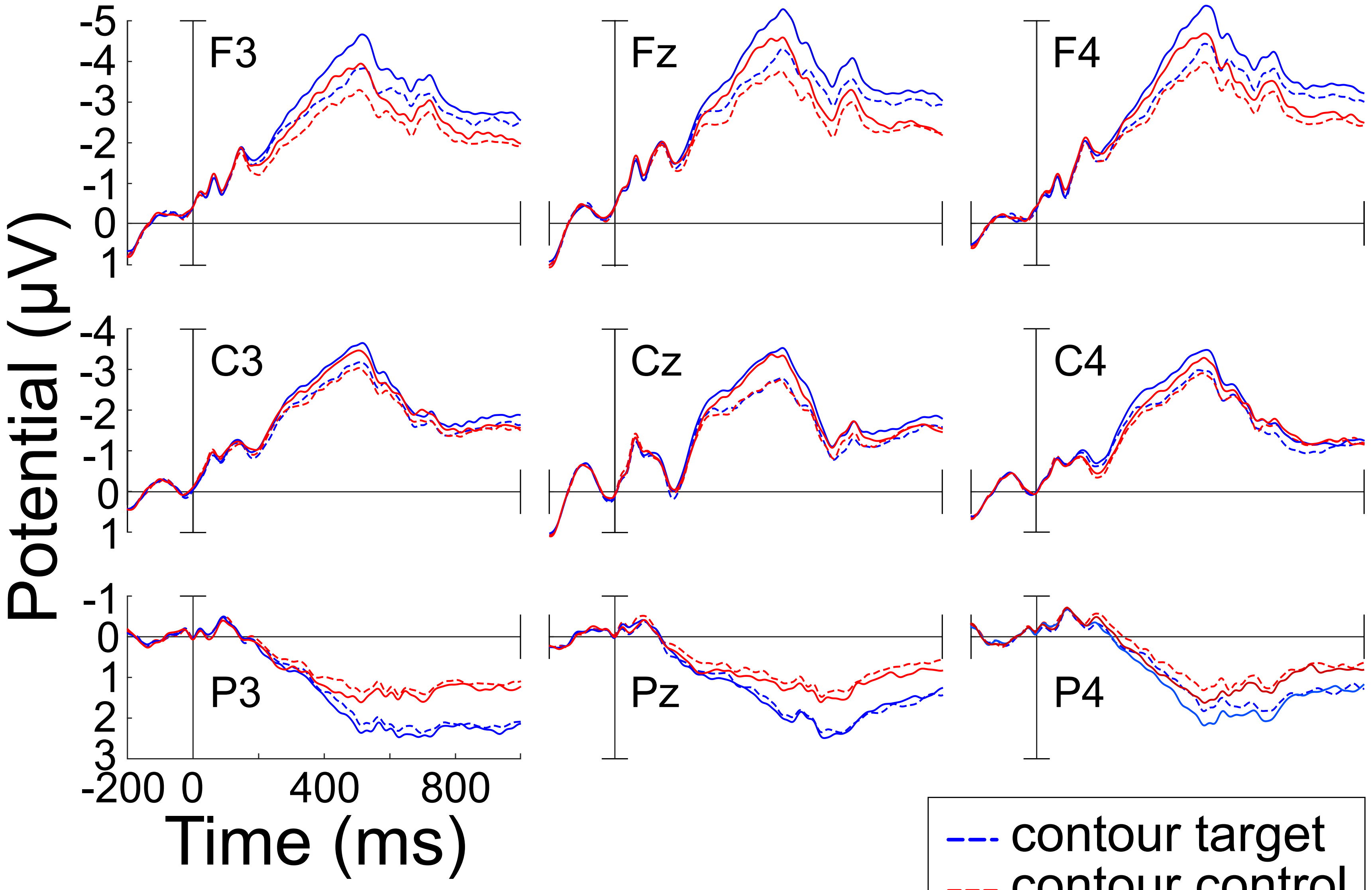

B: non-tonal speakers

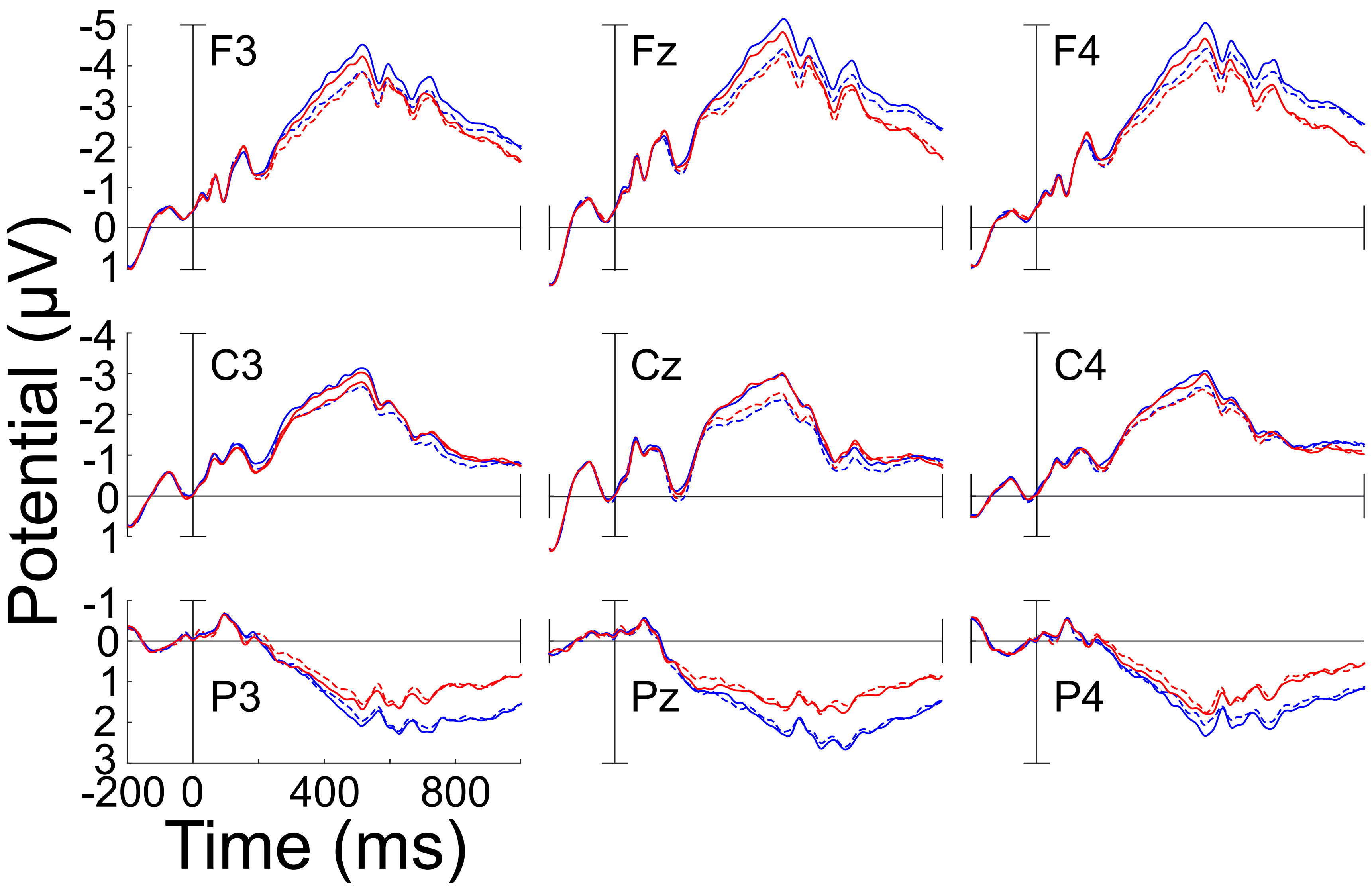

Supplement: Supplementary file 3 — Supplementary Information S3 Six electrode plots illustrating the anterior negativity. Divided by learner group [file PSYP-59-e14042-s001.pdf]
